# Sleep Deprivation Alters the Pituitary Stress Transcriptome in Male and Female Mice

**DOI:** 10.3389/fendo.2019.00676

**Published:** 2019-10-09

**Authors:** Mario G. Oyola, Elizabeth A. Shupe, Anthony R. Soltis, Gauthaman Sukumar, Marcelo Paez-Pereda, Darwin O. Larco, Matthew D. Wilkerson, Stephen Rothwell, Clifton L. Dalgard, T. John Wu

**Affiliations:** ^1^Department of Obstetrics and Gynecology, Uniformed Services University of the Health Sciences, Bethesda, MD, United States; ^2^Center for Neuroscience and Regenerative Medicine, Uniformed Services University of the Health Sciences, Bethesda, MD, United States; ^3^Henry M. Jackson Foundation for the Advancement of Military Medicine, Bethesda, MD, United States; ^4^Collaborative Health Initiative Research Program, Uniformed Services University of the Health Sciences, Bethesda, MD, United States; ^5^Department of Translational Research in Psychiatry, Max Planck Institute of Psychiatry, Munich, Germany; ^6^Department of Anatomy, Physiology and Genetics, Uniformed Services University of the Health Sciences, Bethesda, MD, United States

**Keywords:** pituitary gland, transcriptome, sleep deprivation, stress response, sex differences, hypothalamic-pituitary-adrenal axis, RNA-seq, acute stress

## Abstract

Poor sleep hygiene is a growing problem, with detrimental effects on many biological systems. The pituitary gland plays a crucial role in the regulation of sleep and the stress response, and its dysfunction leads to sleep-related disorders. However, the interaction between these critical functions remains unclear. Thus, we performed a comparative, whole-transcriptome, analysis to identify stress-induced genes and relevant pathways that may be affected by sleep deprivation. One day following 12 h of Paradoxical Sleep Deprivation (PSD), mice were restrained for 20 min. Gene expression changes in the pituitary were assessed via RNA-Seq and Gene Ontology in PSD and/or restrained groups compared to controls. We show that restraint triggers transcriptional responses involved in hormone secretion, the glucocorticoid response, and apoptosis in both sexes, with 285 differentially expressed genes in females and 93 in males. When PSD preceded restraint stress, the numbers of differentially expressed genes increased to 613 in females and 580 in males. The pituitary transcriptome of restraint+PSD animals was enriched for microglia and macrophage proliferation, cellular response to corticosteroids, and apoptosis, among others. Finally, we identify sex-specific differences in restraint-induced genes following PSD. These findings provide genetic targets to consider when studying sleep and the response to stress.

## Introduction

Sleep quantity and quality are constantly threatened by shift work, a fast pace 24 h economy, lifestyle, and many psychological stressors endemic to our modern society (1). It is well-known that sleep is vital for the survival of virtually every advanced organism, and that lack thereof threatens well-being and results in dysregulation of the stress system. It is not uncommon for societal pressures to compromise sleep time and quality by extending work hours late into the night to meet rising productivity and scheduling demands. How might this night of sleep deprivation affect the way that one's body responds to the subsequent day's stressors? Sleep deprivation itself is a stressful experience, priming the body to return to homeostasis by counteracting its effects using several mechanisms (2), most of which are unknown. What is known, however, is that lack of sleep has profound negative consequences on metabolism, the immune response, memory, and survival (3–5). Furthermore, a recent work in humans discovered that just one night of sleep deprivation increases the accumulation of proteins associated with Alzheimer's disease risk (3). Therefore, identifying molecular changes triggered by sleep deprivation that could ultimately lead to disease is imperative for the development of better diagnostic tools and targeted interventional therapies.

Sleep and stress tightly regulate one another, sharing neuronal pathways in the brain that ultimately command the peripheral nervous system to adapt to change. Upon a real or perceived threat, the brain initiates a series of peptide releases that ultimately result in peripheral hormonal signaling (6). This intricate system of communication is called the hypothalamic-pituitary-adrenal (HPA) axis. It begins with the hypothalamus secreting corticotropin releasing factor (CRF) into the hypophyseal portal circulation, which gets detected by corticotrophs in the anterior pituitary gland and prompts its release of adrenocorticotropic hormone (ACTH) into peripheral circulation. ACTH signals the production and release of glucocorticoids from the adrenal gland. Glucocorticoids, in return, display their effects on virtually all body systems and tissues, including the reproductive, cardiovascular, respiratory, integumentary, immune, and nervous systems (7). The specific effects are determined by the chronicity and temporality of circulating glucocorticoids, with some effects being beneficial, and others detrimental.

The pituitary gland plays an integral role in hormonal communication with the rest of the body. Acting as the liaison between brain and systemic organs, it not only plays this central role in the regulation of stress circuitry, but also in the reproductive axis, growth signaling, metabolism, and fluid management through its release of other hormones, including thyrotropin, prolactin, growth hormone, gonadotropins, vasopressin, and oxytocin (8, 9). Based on this crucial role, we examined how paradoxical sleep deprivation (PSD) alters the pituitary gland's transcriptional profile and how PSD impacts stress responses in male and female mice. This model is designed to help map out changes in the transcriptional profile of the pituitary triggered by a night of sleep deprivation. Knowing the roles of various genes and how they interact to regulate biological processes, we can correlate expression levels with predicted functions and biological processes, thus interpreting how the pituitary responds to acute sleep deprivation to make organisms adaptable to subsequent challenging events.

## Methods

### Animals

**Figure 1:**
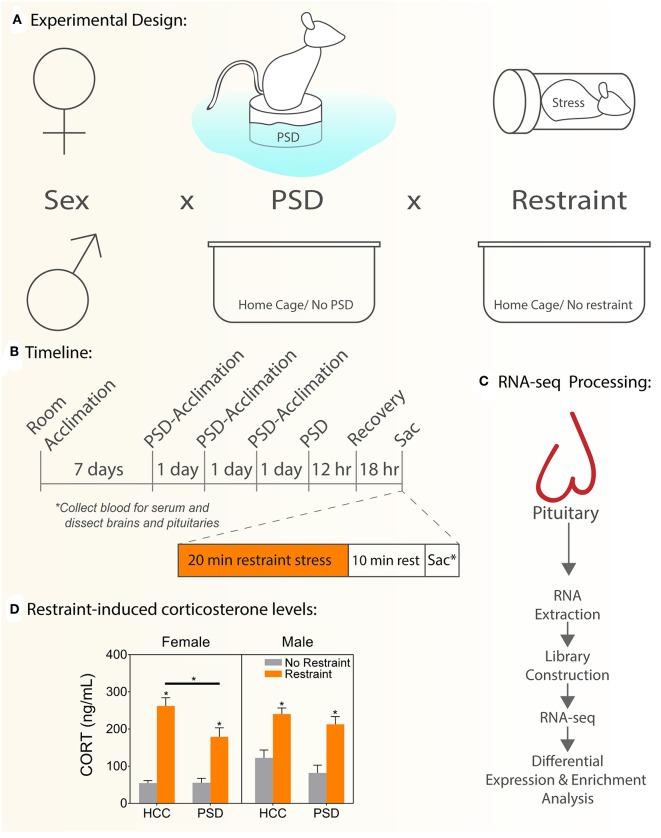
Schematic experimental diagram. **(A)** Comparisons performed: sex (female, male) vs. PSD (sleep restriction, no sleep restriction) vs. restraint stress (restraint, no restraint). **(B)** Schematic representing experimental timeline used to administer paradoxical sleep deprivation, restraint stress, and tissue collection. **(C)** Experimental flow to generate and manage RNAseq data. **(D)** Serum CORT levels. HCC, home cage controls; PSD, paradoxical sleep deprivation; CORT, corticosterone. *p < 0.05. Data are expressed as mean percentage ± SEM.

Male and female C57BL/6J mice, aged 8 weeks old upon arrival, were purchased from The Jackson Laboratory (Bar Harbor, ME) and randomly assigned to each experimental group [No Restraint, No PSD, Restrain Stress, and PSD (Figure 1A)]. All animals were housed by sex in groups of four animals per cage and maintained on a reverse 12 h/12 h lighting cycle (lights on 0700–1900 h) under controlled temperature conditions (22 ± 1°C) with *ad libitum* access to standard rodent chow and water throughout the experimental timeline. Handling and care of animals were conducted in accordance with the National Institutes of Health Guide for Care and Use of Laboratory Animals and approved by the Institutional Animal Care and Use Committee at the Uniformed Services University of the Health Sciences (USUHS), in Bethesda, Maryland.

### Paradoxical Sleep Deprivation (PSD) Protocol

The modified multiple platform method was adapted from previous work to induce PSD while minimizing stress due to social isolation or movement restriction and preserving existing social hierarchies established in the home cage (10–12). This model induces impairment of sleep by producing significant deficits in rapid-eye movement (REM) sleep and partial deficits in non-REM (NREM) sleep (13, 14). Animals that begin to enter the REM stage of sleep, characterized by muscle atonia, are awakened when their snouts touch the water.

PSD tanks were constructed by Stoelting Company (Wood Dale, IL) and engineered of clear acrylic (430 mm length × 260 mm width × 375 mm height) covered with dark paper to minimize visibility of the outside environment. The interior of each tank contained 12 cylindrical columns (145 mm height × 30 mm diameter), one built-in food hopper, and one water bottle. Four socially-familiar cage mates were tested per 12-column tank. The columns were submerged in room-temperature water up to 5–10 mm below the top surface. All animals, regardless of group assignment, were adapted to the sleep deprivation procedure between 0800 and 1200 h for 1 h daily for 3 consecutive days prior to testing (Figure 1B). The purpose of this acclimation period was to habituate animals to the procedure and to attenuate stress associated with exposure to a novel environment (10, 12). On the day of the experimental procedure, mice were placed onto the columns 2–3 h after the onset of the light cycle and sleep-deprived for a period of 12 h. Control animals remained in their home cages in the same room as PSD mice.

### Tissue Collection

Mice were euthanized via carbon dioxide inhalation and rapid decapitation during the nadir of the diurnal CORT cycle 18 h following the end of the sleep deprivation period. To test the effect of overnight sleep deprivation on the neuroendocrine stress response, half of the subjects were restrained for 20 min in flat-bottom, Plexiglas rodent restrainers (3.8 cm width × 9.5 cm length; Plas-Labs, Inc., Lansing, MI). Animals were euthanized 10 min after the end of restraint. Trunk blood and pituitary gland were harvested. Trunk blood was centrifuged at 6,000 *g* at 4°C for 10 min, and serum was harvested in a fresh tube. All samples were stored at−80°C until use (Figures 1B,C).

### Hormone Assay

Corticosterone levels were measured in blood serum using a commercially available ELISA (catalog no. K014; Arbor Assays, Ann Arbor, MI), following the manufacturer's instructions. Samples (*n* = 7–8/group) were diluted to a final concentration of 1:150 and analyzed in duplicate. Changes in binding were measured using a plate reader (Victor V3; PerkinElmer, Waltham, MA), which read absorbance at a wavelength of 450 nm. Values were obtained by comparison, with a standard curve ranging from 78.125 to 10,000 pg/mL. Intra-assay coefficient of variance was 7.5%, and the minimum detectable CORT concentration was 16.9 pg/mL. Comparisons performed: sex (female, male) vs. PSD (sleep restriction, no sleep restriction) vs. restraint stress (restraint, no restraint).

### RNA Extraction and Purification

Whole pituitary samples (*n* = 3–4/group) were homogenized at room temperature in 500 μL of RiboZol (AMRESCO RiboZol RNA Extraction Reagent, VWR, Radnor, PA, USA) using a sterile 1-mL syringe equipped with a 22-gauge needle tip. Total RNA was isolated using the Direct-zol RNA MiniPrep Plus Kit (Zymo Research, Irvine, CA). A final elution volume of 30 μL of nuclease-free water was added to the spin column and centrifuged at 12,000 × *g* for 1 min. Total RNA concentration and purity ratios were quantified by a NanoDrop Lite Spectrophotometer (Thermo Scientific, Waltham, MA). Purified RNA samples were stored at −80°C.

### Pituitary Transcriptome Profiling by RNA-Seq

RNA integrity was assessed using automated capillary electrophoresis on a Fragment Analyzer (Advanced Analytical). Total RNA input of 500 ng was used for library preparation using the Truseq Stranded mRNA Library Preparation Kit (Illumina, San Diego, CA, USA). Sequencing libraries were quantified by PCR using KAPA Library Quantification Kit for NGS (Kapa, Wilmington, MA, USA) and assessed for size distribution on a Fragment Analyzer. Sequencing libraries were pooled and sequenced on a HiSeq3000 (Illumina) using 150 cycle SBS kit with paired-end reads at 76 bp length (15). cDNA was reversed transcribed using the purified RNA using a commercially available reverse transcription kit (Thermo Scientific Maxima Reverse transcription kit; catalog no. K1671; Thermo Fisher Scientific).

### Real-Time Quantitative PCR Verification

Pituitary mRNA expression of genes known to be affected by the selected sleep and stress challenges were measured by quantitative PCR, following previously established protocols (16), were used to verify differentially expressed genes. Comparisons performed: Sex (female, male) vs. PSD (sleep restriction, no sleep restriction) vs. restraint stress (restraint, no restraint). In brief, 2 ng cDNA template was amplified using iQ SYBR Green Supermix (Bio-Rad, Hercules, CA). Primer sequences [pro-opiomelanocortin (POMC), prohormone convertase 2 (Pcsk2), CRF receptor subtype 1 (CRFR1), mineralocorticoid (MR) and glucocorticoid (GR) receptors, corticotropin-releasing factor binding protein (Crfbp), and TATA-box binding protein (TBP)] were designed to have a common annealing temperature of 60°C using the National Center for Biotechnology Information Primer blast (Table S1) (16). Each run consisted of an initial denaturation (3 min at 95°C), followed by 40 cycles that included denaturation (10 s at 95°C), annealing (30 s at 60°C), and extension (30 s at 72°C) using the CFX Connect Real-Time System (Bio-Rad). Melt-curve analysis was performed after each run to ensure a single amplicon. All data were normalized to TATA box-binding protein and expressed relative to the control group (Supplementary Figure 1). The 2^−ΔΔ*CT*^ method was used for analysis. All samples (*n* = 3–4/group) were analyzed in duplicate. Except for the male Crhbp expression, all qRT-PCR relative expression patterns were similar to the gene counts patterns observed on the RNA-seq data.

### Statistical Analysis

CORT and qRT-PCR data were analyzed using three-way ANOVA (GraphPad Software, La Jolla, CA). Sidak's (CORT) and Tukey's (qRT-PCR) tests were used for *post hoc* analysis when a significant effect was present. *p* < 0.05 was considered significant.

### RNA-Seq Alignment, Quantification, and Differential Expression Analysis

Raw paired-end sequencing reads were aligned to the mouse reference genome (mm10) using MapSplice (17) (version 2.2.1). Gene read counts against UCSC mouse gene models (obtained on February 21, 2016) were calculated by HTSeq (18) (version 0.9.1) with the mode parameter set to “intersection-nonempty.” For transcripts per million (TPM) calculations, the average of all transcript lengths corresponding to the same gene were used for gene length factors and only protein-coding genes (identified by the presence of at least one annotated CDS element in its gene model) were considered in “per million” normalization factor calculations. Read alignment statistics and sample quality features were calculated with SAMtools (19) and RSeQC (20). Sequencing quality was verified by manual inspection of sample-wise characteristics: total reads, mapping percentages, pairing percentages, transcript integrity number (TIN) (21), 5′ to 3′ gene body read coverage slopes, and ribosomal RNA content.

Differential gene expression was estimated with DESeq2 (22) (version 1.16.1). For the main analysis (i.e., using all samples), we used the following contrast model to simultaneously model all known experimental main effects: *y* ~ *sex* + *psd* + *restraint stress* + *psd:restraint stress*. The reference levels for the main treatment effects “sleep” and “stress” were set to “home cage control” and “no stress,” respectively. In analyses considering male and female mice separately, the “sex” term was dropped while retaining the same contrast definition for the main treatment effects. Per contrast, genes with mean TPM expression levels greater than or equal to one across the cohort were considered differentially expressed if their estimated adjusted *p*-values were ≤0.05 and if the absolute value of their log_2_ fold-changes following shrinkage (i.e., using the lfcShrink function in DESeq2) were ≥0.322.

### Gene Ontology and Clustering Analysis

Gene ontology (GO) term enrichments were calculated by hypergeometric tests of foreground differentially expressed gene term counts against a background of all expressed genes (mean TPM ≥ 1). Terms with ≤1,000 total genes in the GO database downloaded on October 2, 2017 were considered. *P*-values were adjusted using the Benjamini-Hochberg FDR procedure and terms with FDR < 0.1 were deemed significant. Hierarchical clustering was performed in Python using SciPy library functions (https://www.scipy.org/) and displayed using Matplotlib (23). Graphical representations of GO term enrichment results were generated in Cytoscape (24).

## Results

### PSD Blunts Neuroendocrine Response to Stress in a Sex-Dependent Manner

To determine the effect of PSD on the neuroendocrine response to stress, we measured CORT levels 18 h following 12 h of PSD (Figure 1D). In females, there was an interaction effect between PSD and restraint [*F*_(1, 26)_ = 7.54; *p* = 0.032]. Significant main effects of PSD and restraint were also detected in females [*F*_(1, 26)_ = 4.88; *p* = 0.036 and *F*_(1, 26)_ = 79.3; *p* < 0.0001, respectively]. Interaction analysis using Sidak's multiple comparison test revealed the usual significant increase in CORT following restraint regardless of PSD treatment (*p* < 0.001). However, this response to restraint was significantly blunted in the females exposed to PSD (*p* < 0.018). Males, however, did not show an interaction effect (PSD vs. restraint). Similar to the pattern detected in females, a main effect of PSD [*F*_(1, 51)_ = 7.54; *p* = 0.008] and restraint [*F*_(3, 51)_ = 39.05; *p* < 0.0001] was detected in males.

### Stress-Related Pituitary Transcriptome Is Altered Following PSD

**Figure 2:**
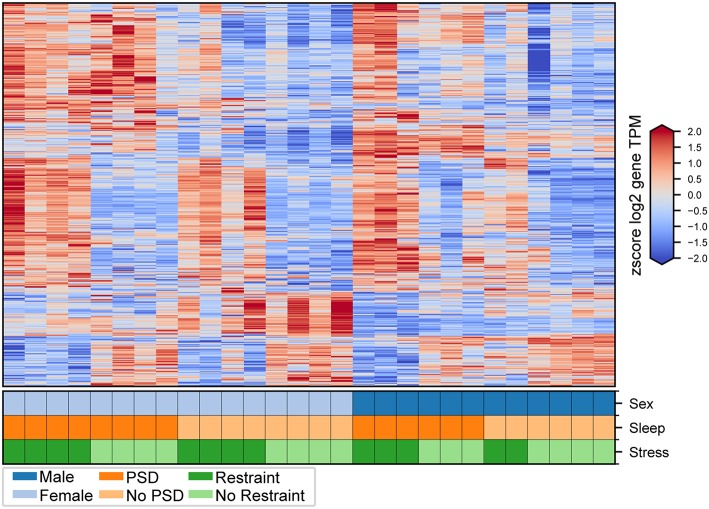
Transcriptional changes induced by PSD and restraint. Expression levels for genes consistently differentially expressed in male and female mice following PSD alone (412 genes), restraint alone (231 genes), and combination PSD + restraint (963 genes). Heatmap values are z-scored log_2_ TPM gene expression levels. The lower panel indicates mouse sex (top row, male or female), PSD (middle row, PSD or no PSD), and restraint (bottom row, restraint or no restraint).

**Figure 3:**
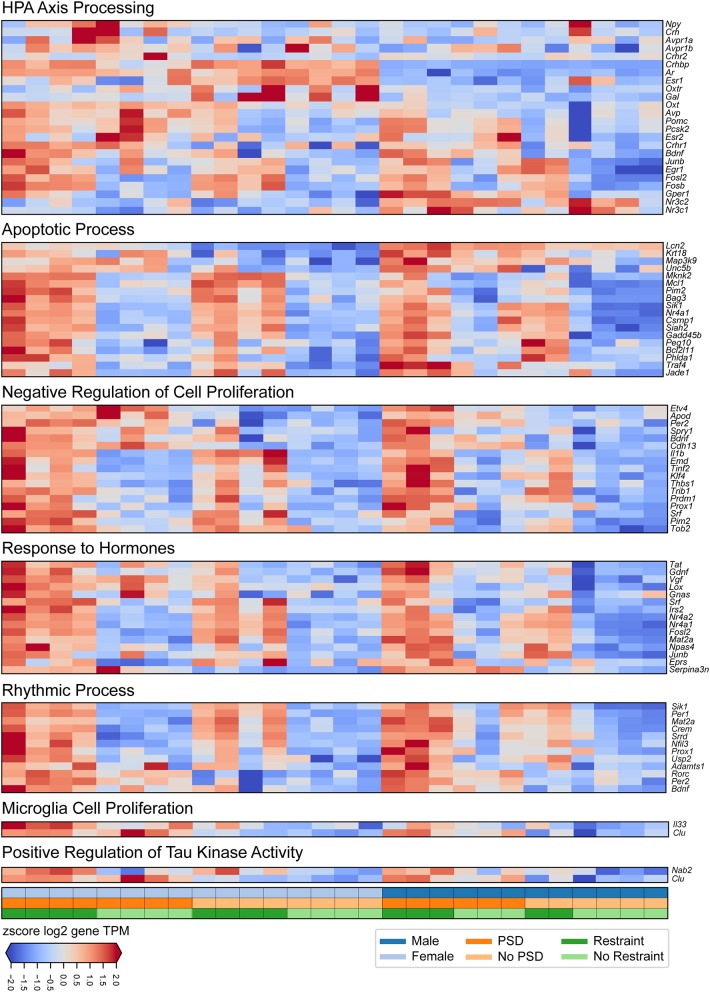
Transcriptional changes induced by PSD and restraint involved in specific biological processes. The panels show (from top to bottom) expression signatures of differentially expressed genes involved in HPA-axis processing, apoptotic processes, negative regulation of cell proliferation, response to hormones, rhythmic processes, microglia cell proliferation, and positive regulation of tau kinase activity. The values displayed in each heatmap are z-scored log_2_ TPM gene expression levels. The lower panel indicates mouse sex (top row, male or female), PSD (middle row, PSD or no PSD), and restraint (bottom row, restraint or no restraint).

We first interrogated pituitary transcriptional changes involved in the neuroendocrine response to stress. Second, we aimed to interrogate how this transcriptional signature is affected by 12 h of PSD. We identified consistent transcriptional changes between males and females (Figures 2, 3). Results in males and females are discussed separately. We used this method to assess sex-specific effects driven by PSD and/or restraint stress. Finally, we discuss the differential expression analysis considering all mice (i.e., both males and females from all treatment groups) in a single model.

#### Females

**Figure 4:**
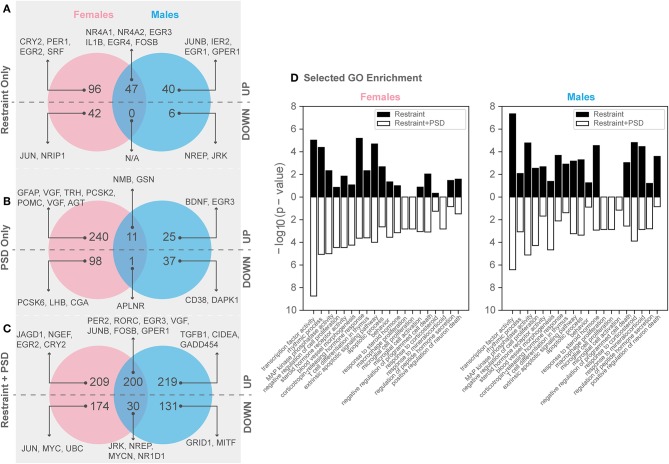
Genes significantly altered by PSD and restraint. **(A)** Restraint only genes, **(B)** PSD only genes, **(C)** Restraint only vs. restraint and PSD genes, **(D)** Selected GO enrichment for all DEGs in restraint only (black) and restraint + PSD (white) Left: Females: Restraint_CageControl vs. Restraint_PSD, Right : Males: Restraint_CageControl vs. Restraint_PSD.

**Figure 5:**
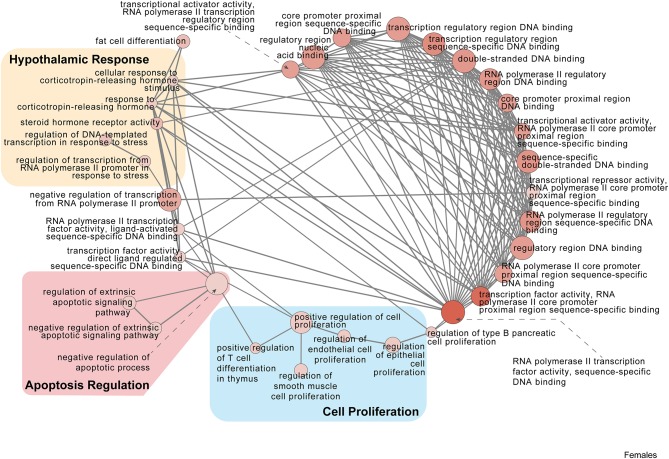
Female PSD and restraint stress-induced common biological processes. This functional annotation network shows the biological and molecular functions of pathways altered by the combination of PSD and restraint stress when compared to the control groups (no PSD, no restraint). The node size reflects the size of the gene set (i.e., the number of DEGs in that GO term) altered by each treatment. Node color is continuously shaded based on the term significance level [as –log_10_(*p*-value)]. Edges are created based on overlap of the genes contained in the terms. Edge width is continuously scaled based on the edge weight (i.e., the strength of the overlap between the genes in two gene sets). The genes used to generate these figures were first selected in an unbiased fashion by the DEG analysis and then subsequently selected based on uncovered GO/pathway analysis.

**Figure 6:**
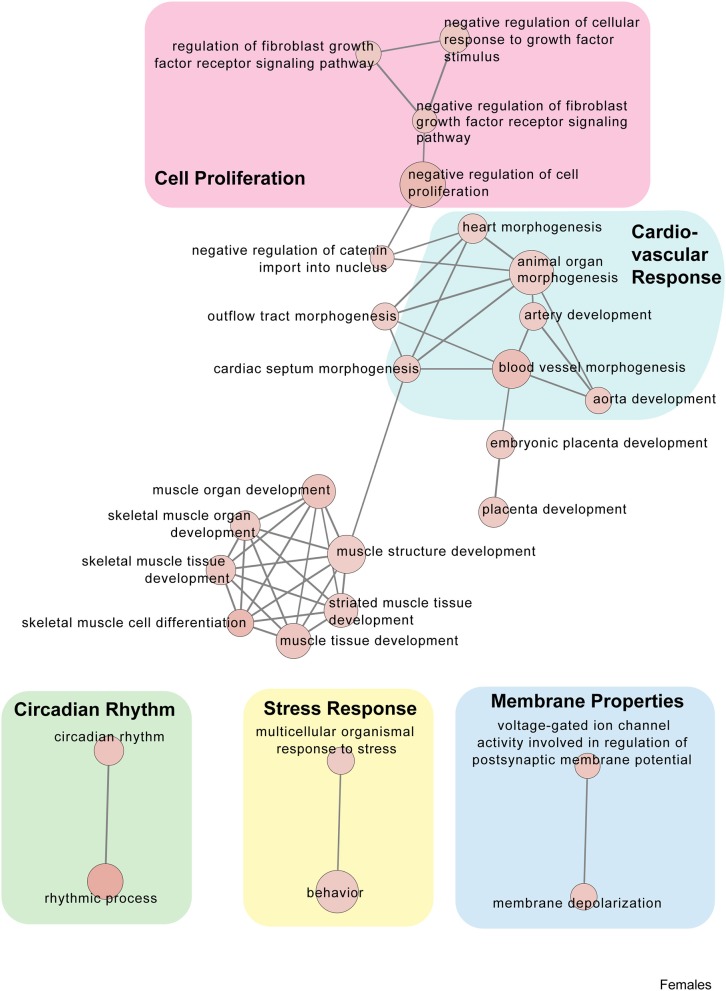
Female PSD and restraint stress-induced common biological processes. This functional annotation network shows the biological and molecular functions of pathways altered by the combination of PSD and restraint stress when compared to the control groups (no PSD, no restraint). The node size reflects the size of the gene set (i.e., the number of DEGs in that GO term) altered by each treatment. Node color is continuously shaded based on the term significance level [as –log_10_(*p*-value)]. Edges are created based on overlap of the genes contained in the terms. Edge width is continuously scaled based on the edge weight (i.e., the strength of the overlap between the genes in two gene sets). The genes used to generate these figures were first selected in an unbiased fashion by the DEG analysis and then subsequently selected based on uncovered GO/pathway analysis.

Twenty minutes of restraint stress elicited widespread changes to the pituitary transcriptome compared to home cage controls, with 285 differentially expressed genes in female mice (143 upregulated and 42 downregulated) (Figure 4). In females, the restraint stress-associated transcriptome signature was enriched for established stress response actions involved in hormone secretion and rhythmic and apoptotic processes (Figures 4–6, Table S2). We detected a particular differential signature of 350 genes (251 upregulated and 99 downregulated) in female mice exposed to PSD as compared to home cage controls (Figure 4, Supplementary Dataset 1). Figures 5, 6 show functional annotation networks for biological and molecular pathways altered by the combination of PSD and restraint stress when compared to the control groups (no PSD, no restraint). These graphs are based on GO term enrichments for all DEGs (i.e., up + down) in Restraint + PSD animals vs. females only. The pituitary transcriptome of PSD females was enriched for canonical cellular activities involved in homeostasis, differentiation, and behavior, among others. The largest number of differentially expressed genes, 613, was detected when the animals were sleep deprived 18 h prior to the restraint challenge. Of these genes, 409 were upregulated, while 204 were downregulated. Functional enrichment analysis for these 613 differentially expressed genes showed that the added effect of PSD and restraint challenge affects a range of processes including circadian rhythms, response to CRF, cellular differentiation, and negative regulation of many key biological functions (Figures 4A–D, Table S2).

#### Males

**Figure 7:**
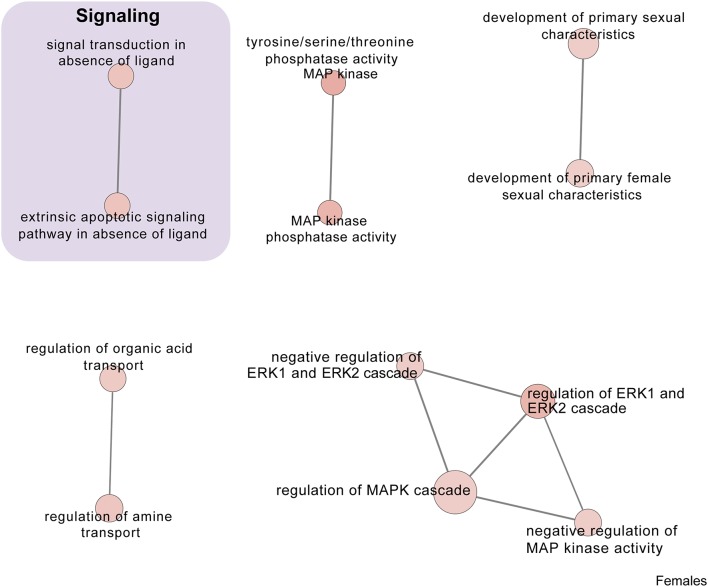
Male PSD and restraint stress-induced common biological processes. This functional annotation network shows the biological and molecular functions of pathways altered by the combination of PSD and restraint stress when compared to the control groups (no PSD, no restraint). The node size reflects the size of the gene set (i.e., the number of DEGs in that GO term) altered by each treatment. Node color is continuously shaded based on the term significance level [as –log_10_(*p*-value)]. Edges are created based on overlap of the genes contained in the terms. Edge width is continuously scaled based on the edge weight (i.e., the strength of the overlap between the genes in two gene sets). The genes used to generate these figures were first selected in an unbiased fashion by the DEG analysis and then subsequently selected based on uncovered GO/pathway analysis.

**Figure 8:**
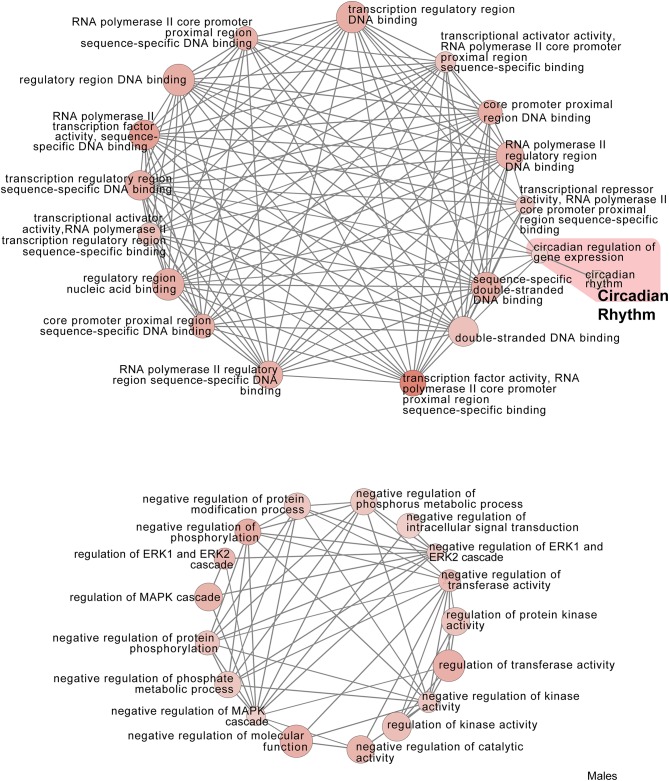
Male PSD and restraint stress-induced common biological processes. This functional annotation network shows the biological and molecular functions of pathways altered by the combination of PSD and restraint stress when compared to the control groups (no PSD, no restraint). The node size reflects the size of the gene set (i.e., the number of DEGs in that GO term) altered by each treatment. Node color is continuously shaded based on the term significance level [as –log_10_(*p*-value)]. Edges are created based on overlap of the genes contained in the terms. Edge width is continuously scaled based on the edge weight (i.e., the strength of the overlap between the genes in two gene sets). The genes used to generate these figures were first selected in an unbiased fashion by the DEG analysis and then subsequently selected based on uncovered GO/pathway analysis.

**Figure 9:**
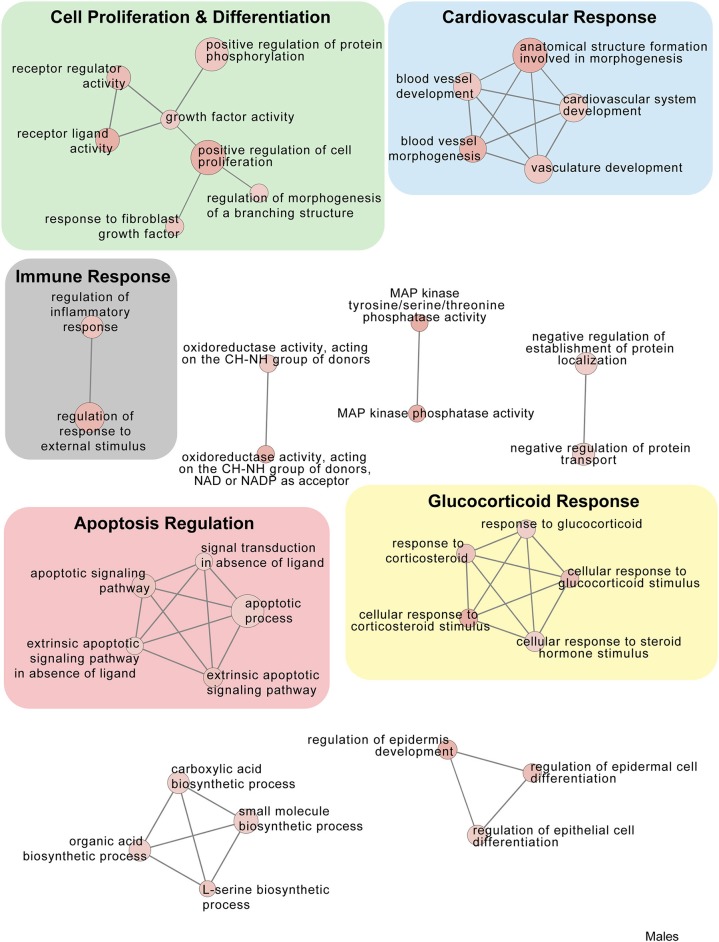
Male PSD and restraint stress-induced common biological processes. This functional annotation network shows the biological and molecular functions of pathways altered by the combination of PSD and restraint stress when compared to the control groups (no PSD, no restraint). The node size reflects the size of the gene set (i.e., the number of DEGs in that GO term) altered by each treatment. Node color is continuously shaded based on the term significance level [as -log10(*p*-value)]. Edges are created based on overlap of the genes contained in the terms. Edge width is continuously scaled based on the edge weight (i.e., the strength of the overlap between the genes in two gene sets). The genes used to generate these figures were first selected in an unbiased fashion by the DEG analysis and then subsequently selected based on uncovered GO/pathway analysis.

In males, restraint stress also induced extensive changes to the pituitary transcriptome compared to home cage controls, with 93 differentially expressed genes (86 up-regulated and 6 down-regulated). A short period of restraint stress drastically changed the male pituitary transcriptome, targeting genes involved in the regulation of the corticosteroid response, programmed cell death, and the CRF response, among others (Figures 3, 4, 7–9, Tables S3, S4). Figures 7–9 show functional annotation networks for biological and molecular pathways altered by the combination of PSD and restraint stress when compared to the control groups (no PSD, no restraint). These graphs are based on GO term enrichments for all DEGs (i.e., up + down) in Restraint + PSD animals vs. males only. Following PSD, 74 genes were differentially expressed (36 genes upregulated and 38 downregulated) in the pituitary of male mice (Figure 2, Supplementary Dataset 1). Among the processes regulated by these 74 differentially expressed genes were lymphocyte regulation and the response to interferon gamma and cytokines (Figure 4B, Supplementary Dataset 1). The most substantial change in the pituitary transcriptome was detected when the animals were sleep deprived for 12 h and challenged with restraint stress 18 h later. This additive effect resulted in 580 differentially expressed genes (419 upregulated and 161 downregulated). Following a functional enrichment analysis, we detected that the genes involved in this compound effect are known to regulate microglia and macrophage proliferation, cellular response to corticosteroids, the inflammatory response, cell death, and tau kinase activity (Figures 4C,D, 7–9, Table S3).

### Females and Males: Conserved Genes and Sex-Differences in the Sleep Deprived Challenged Response to Stress

The response to restraint stress was dampened in males and females exposed to PSD, but this effect was more pronounced in females. Similarly, the pituitary transcriptome of males and females was strikingly similar following restraint and PSD. Male and female mice exposed to restraint have 47 genes in common [138 unique in females, 46 unique in males (Supplementary Dataset 1)]. Differentially expressed genes following restraint stress, common in both, males and females, are involved in response to CRF, apoptotic processes, and muscle development (Figure 4A, Supplementary Dataset 1, Tables S2–S4). This number of differentially expressed genes was even smaller in animals exposed to PSD (338 unique in females, 62 unique in males, and 12 in common). Negative regulation of peptide activity, regulation of angiogenesis, and homeostasis are among the processes controlled by the differentially expressed genes common in both sexes following sleep deprivation. However, when both sexes were sleep deprived prior to the restraint challenge, the similarity between differentially expressed genes was close to 40% (383 unique in females, 350 unique in males, and 230 in common) (Figure 4C). These genes are involved in the regulation of circadian rhythms, apoptosis, and immune and mineralocorticoid response, among many other processes (see Supplementary Dataset 1, Tables S2–S4).

We additionally performed differential expression analysis considering all mice (i.e., both males and females from all treatment groups) in a single model to identify consistent transcriptional changes resulting from restraint stress, PSD, and the combination of the two challenges (see Methods). This analysis first identified 1,918 genes significantly different between mice based on background sex alone [927 up-regulated and 991 down-regulated in females compared to males (Supplementary Figure 2)]. In terms of consistent treatment effects, this analysis uncovered 213 genes altered by restraint stress alone, 200 genes altered by PSD alone, and 728 genes affected by both challenges (Supplementary Dataset 2).

## Discussion

The biological response to stress is key for survival and this response is affected by sleep deprivation. To our knowledge, this is the first work showing the stress-induced transcriptome of the male and female mouse pituitary, illustrating common transcriptional landscapes unique and common between sexes. Furthermore, we study the effects of sleep deprivation inflicts on the response to stress. In this study, we show that 12 h of PSD is enough to affect this response even 1 day following the sleep deprivation episode. Using a combination of stress hormone detection and transcriptomics, we show that PSD dampens the response to restraint stress. As in many other aspects regarding the HPA axis response to stress, the observed effects following PSD were more prominent in female than male mice. These observations were further confirmed at the pituitary level, where we detected marked differences in the whole transcriptome of males and females following restraint stress. Although many of the differentially expressed genes were common between males and females exposed to restraint stress, the number of unique genes was striking. Interestingly, the restraint stress-induced transcriptome of males and females exposed to PSD was drastically different from the control group, indicating the marked effect that PSD could have on the whole transcriptome.

Mutually highly interconnected systems organize the regulation of stress and sleep. These systems affect metabolism, HPA and hypothalamic-pituitary-gonadal (HPG) axes, and virtually every system in the body (25). Not surprisingly, dysregulation of either or both of these biological functions results in a number of metabolic and neuropsychiatric disorders (26). Sleep quantity and quality are threatened in our society. Thus, the number of pathologies associated with poor sleep hygiene are on the rise, and it should be of concern to the health agencies. In this work, we investigated the effects of 12 h of PSD on the pituitary transcriptome. It is important to note that we aim to elucidate the effects observed the day following sleep deprivation, targeting its long-lasting properties and designing the experiment to increase the translational value. To the extent of our knowledge, this is the first study interrogating the effects of sleep deprivation at the pituitary level, and the first study comparing the male and female pituitary transcriptome after such intervention [for review see (27)].

We collected male and female pituitary glands 18 h following a period of 12 h of PSD to interrogate the effects of sleep deprivation on a subsequent stress challenge. One of the most surprising results from these experiments was the evident dampening of the response to stress in animals exposed to PSD. Although the sleep deprivation was induced 18 h prior to the restraint stress challenge, restrain-induced CORT levels in female mice were lower than the controls. These data suggest that 12 h of PSD exerts effects on the HPA axis that prevail until the next day, thus atrophying the neuroendocrine response to stress.

Every organism must adapt to changes. The brain is the master organ regulating the response to stress, via the perception of the actual stressor and orchestrating an equivalent response to reach homeostasis (28). Failure to adapt to these changes could lead to pathophysiology. To deal with the imminent threat, whether real or perceived, the brain and body recruit the neuroendocrine, autonomic, metabolic, and immune systems (29). In the present study, we show how the pituitary, the initial component of the neuroendocrine response to stress, remodels its stress-induced transcriptome to adapt to a previous stressor (i.e., 12 h of PSD). Our finding that PSD decreases the amount of restraint-induced CORT is unique, as many studies looking at CORT levels immediately after PSD report pronounced increases in this stress hormone (30, 31). Only a few studies have looked at the effects of PSD on a subsequent stressor. Merlo et al., showed that the response to 30 min restraint stress 4 h following 48 h of sleep deprivation results in decreased ACTH levels in male rats, however, CORT levels were back to baseline at this point (2). Of note, they used a slowly rotating wheel to inflict the sleep deprivation. While this is a completely different sleep deprivation model, they also detected a persistent effect of sleep deprivation on the neuroendocrine stress response. It would be interesting to know if a more robust effect would be detected in females, as in the present study.

The pituitary gland sits in an optimal place to interrogate how the brain responds to stressors, signaling through hormones, and how the peripheral tissues react to these signals (feedback). This “master gland” is divided into three main parts: intermediate, adenohypophysis (anterior), and neurohypophysis (posterior). It is important to consider the composition of this mixed origin gland. While, the main releasing hormone cells driving downstream effects reside in the adenohypophysis, PSD may have an effect in the other parts of the pituitary. Thus, future studies are needed to dissect division-dependent effects. A potential way to narrow down the particular function of each of the cells residing in each of the pituitary division might be using a cell sorting technique followed by RNA-seq. Very few studies have mined the gene expression differences in the pituitary, perhaps because of limitations in technology. Nishida et al. gave us good clues about gene expression information indicating sex differences in the pituitary (32), which we complimented in the current work looking at the whole pituitary transcriptome. However, in terms of the response to stress, the expression of sexually dimorphic genes in the pituitary remains to be fully uncovered. Therefore, we used RNA-seq and differential expression analysis to detect genes whose expression is altered following restraint stress, and to detect how this expression further change when animals are sleep deprived. These genes were further dissected using GO pathway analysis to detect which biological processes are targeted by these differentially expressed gene. It is also important to be aware of the powerful effects of sex hormones and many other sex-dependent factors that may have a considerable impact on the transcriptome, and this is one of the reasons we performed two different comparisons: sex by itself (males only, females only), revealing sex-specific genes and another in which sex was used as a covariant.

The restraint-induced differential expression pattern between female and males show considerable overlap. However, we detected a large number of restraint-induced genes that are unique to each sex, perhaps explaining the sex differences in stress response. In a previous work looking at sex differences in the pituitary transcriptome using serial analysis gene expression, 43 differentially expressed transcripts by gender were detected (32). The differentially expressed genes reported by that group are involved in cell signaling, mitochondrial transport, chromatin structure and remodeling, and cell differentiation among others. Here, we report that 47 genes are differentially expressed in both males and females following 20 min of restraint stress. Among these genes *Fosl2, Per1*, and *Cry2* were upregulated in females only following restraint stress. In males, the pattern was quite different. *Junb1, Egr1*, and *Gper1* were some of the genes upregulated in males. While all of these genes are involved in regulation of the stress response, their mechanisms of action are different (33–37).

Restraint stress also resulted in overlapping differentially expressed genes in both male and females. Some of the ones involved in the stress response are *Il1b, Egr3*, and *Fosb* (38–40). Together, the common restraint-induced differentially expressed genes between males and females are involved in the response to CRF, signal response in the absence of ligand, and regulation of the apoptotic signaling pathway. These data suggest that while there are many stress-related genes involved in the evolutionary well-conserved response to stress, the differential expression of genes unique to each sex may be driving the differential activation of this response.

Sleep deprivation exerted dramatic changes to the pituitary transcriptome landscape in male and female mice. However, only 12 differentially expressed genes were shared between male and female mice exposed to PSD. More than 300 genes were unique to females and 62 to males, indicating a large sex differences in the overall gene recruitment regulating sleep deprivation. Among the differentially expressed genes in females, we detected *Vgf, Pomc*, and *Th*. The expression of these genes have been shown to be affected by sleep deprivation (41–43). As previously mentioned, the pituitary sits in a key place serving as gatekeeper of many physiological functions, and thus, it is not surprising that following PSD, we detect striking changes in genes involved in neuronal differentiation, gliogenesis, and behavior regulation. The importance of sleep is highlighted by these GO terms. For example, the need for neuronal differentiation may indicate that sleep deprivation leads to changes in neuronal heath and number that need to be corrected, process that is known to take place during sleep (44, 45). Events like sleep deprivation tend to shift the organism away from homeostasis resulting in the activation of many survival systems including the immune system. Thus, it is not surprising the that genes needed for gliogenesis are activated following PSD, perhaps to increase the number of microglia and astroglia during the recovery period following PSD.

On the other hand, the PSD-induced effects on the male pituitary transcriptome appear different. We detected changes to immune response genes involved in response to interferon-gamma, antigen processing, and regulation of lymphocytes. These data indicated that the effects of PSD are long-lasting, especially in the regulation of the immune system. Similar effects have been reported in several studies, indicating a reduction in the ability of the immune system to respond to challenges following sleep deprivation (46–48). Nevertheless, not many studies addressing changes at the pituitary level, elicited by sleep deprivation, are available. The striking difference detected in the differentially expressed genes induced by PSD among male and female pituitary transcriptome suggests that sex-specific pathways may be recruited to deal with the sleep challenge.

Many reports using different methods to induce sleep deprivation have reported profound effects on the response to stress (2, 13, 49). These reports suggest that the HPA axis response to stress is shaped by sleep hygiene. Our main aim was to elucidate how sleep deprivation could reformat the pituitary transcriptome affecting the way animals respond to subsequent stress challenges. Interestingly, it seems that the transcriptome effects observed by restraint stress on the pituitary genome are amplified when animals are sleep deprived 18 h prior. The amount of differentially expressed genes more than doubled when animals are sleep deprived prior to restraint stress compared with animals that were only restraint-stressed. Additionally, the number of conserved genes between both sexes was significantly higher, suggesting the recruitment of alike systems to deal with these insults. We detected a group differentially expressed genes involved in the stress response including *Per1, Per2, Gepr1, Bdnf*, and *Fosb*. However, the number of genes differentially expressed extended far beyond the regulation of stress. For instance, the number of genes regulating cell death and the immune system was very prevalent. Included in the list of genes involved in programmed cell death we found *Bcl2l11, Unc5b*, and *Mknk2*. Various works have reported an increase in cell death following sleep deprivation. For example, Somarajan and colleagues reported an increase in markers of cell death in adult male rats following 6 days of sleep deprivation (50). However, in our work, we show that the effects of 12 h of sleep deprivation continue to be expressed 18 h after this insult. PSD prior to restraint stress resulted in the differential expression of *Clu, IL33*, and *IL1b*, all of which are involved in the regulation of the immune system (51–54).

Of note, in a recent report in humans, one night of sleep deprivation was sufficient to induce accumulation of proteins associated with Alzheimer's disease (3). In the current work, we detected that *Clu* and *Nab2*, genes involved in the positive regulation of tau-protein kinase activity, are differentially expressed following PSD + restraint stress. These data suggest that changes associated with diseases such as dementia could also be manifested at the pituitary level following sleep deprivation.

The cardiovascular system seems to be a major target affected by the combination of sleep deprivation followed by a stressful challenge as we detected differentially expressed genes involved in the regulation of the circadian system and vasculature development (55). These genes are also involved in blood vessel morphogenesis and development. Together, these data suggested that the combination of these two insults recruit a number of vasculature related genes perhaps to compensate for the high energy demand needed during and following a stressor. Given these data, the logical next series of experiments should explore the effects of longer periods of sleep restriction followed by subsequent stressful challenges. These manipulations might result in physiological changes of a magnitude that the body may not be able to meet, leading to the pathology of the cardiovascular system. This idea is not far from possible given the current knowledge of the effects of stress (56) and sleep deprivation (57) on the cardiovascular system.

In summary, we studied the pituitary transcriptome of male and female mice exposed to 12 h PSD 18 h prior to a stressful challenge. The results indicate marked similarities and differences in the male and female stress-induced transcriptome, as well as in the PSD-induced changes. When PSD preceded the restraint stress challenge, the effects on the pituitary transcriptome were striking. While the male and female PSD + restraint-induced transcriptome was similar, we detected remarkable differences, perhaps indicating different strategies used by each sex to cope with challenges to homeostasis. We hope that these data illuminate future research elucidating how sleep deprivation impacts the vital response to stress and motivate the analysis of male and female subjects when designing experiments.

## Data Availability Statement

The accession number for the RNA-seq raw and processed data reported in this paper is GEO: GSE133334.

## Ethics Statement

The animal study was reviewed and approved by Institutional Animal Care and Use Committee at the Uniformed Services University of the Health Sciences (USUHS), in Bethesda, Maryland.

## Disclosure

The opinions or assertions contained herein are the private ones of the authors and are not to be construed as official or reflecting the views of the Department of Defense or the Uniformed Services University of the Health Sciences. The authors report no conflicts of interest in this work.

## Author Contributions

MO, ES, and TW conceived and planned the experiments. ES carried out the PSD and qRT-PCR. MO, TW, and MP-P contributed to the interpretation of results. AS, MW, and CD performed RNA-sequencing, alignment, quantification, and differential expression analysis. GS generated library. DL and SR helped conceive the original idea. MO wrote the manuscript. All authors provided feedback and provided integral help in generating this manuscript.

### Conflict of Interest

The authors declare that the research was conducted in the absence of any commercial or financial relationships that could be construed as a potential conflict of interest.

## Supplementary Material

Supplementary Figure S1 Validation of gene expression in the pituitary using qRT-PCR. HCC, home cage controls; PSD, paradoxical sleep deprivation; CORT, corticosterone. ^*^*p* < 0.05. Data are expressed as mean percentage ± SEM.

Supplementary Figure S2 Sex-dependent transcriptional changes along with PSD and Restraint induced alterations. Expression levels for genes differentially expressed between male and female mice (1,317 genes). Genes altered following PSD alone (400 genes), restraint alone (184 genes), and combination PSD + restraint (733 genes) are also displayed. Heatmap values are z-scored log_2_ TPM gene expression levels. The lower panel indicates mouse sex (top row, male or female), PSD (middle row, PSD or no PSD), and restraint (bottom row, restraint or no restraint).

Supplementary Table 1 Primer sequences used for real-time qPCR.

Supplementary Table 2 Female: PSD Up.

Supplementary Table 3 Male: PSD Up.

Supplementary Table 4 Male: PSD Down.

Supplementary Dataset 1 Excel workbook contains results from performed analyses considering male and female mice separately, the “sex” term was dropped while retaining the same contrast definition for the main treatment effects. Per contrast, genes with mean TPM expression levels greater than or equal to one across the cohort were considered differentially expressed if their estimated adjusted *p*-values were ≤0.05 and if the absolute value of their log_2_ fold-changes following shrinkage (i.e., using the lfcShrink function in DESeq2) were ≥0.322.

Supplementary Dataset 2 Excel workbook contains results from performed differential expression analysis considering all mice (i.e., both males and females from all treatment groups) in a single model to identify consistent transcriptional changes resulting from restraint stress, PSD, and the combination of the two challenges. Adjusted *p*-values were ≤0.05 and if the absolute value of their log_2_ fold-changes following shrinkage (i.e., using the lfcShrink function in DESeq2) were ≥0.322.
