# Supplementary figures and images for: Sleep Deprivation Alters the Pituitary Stress Transcriptome in Male and Female Mice
Source: Front Endocrinol (Lausanne). 2019 Oct 9;10:676. doi: 10.3389/fendo.2019.00676 (PMC6794367; doi:10.3389/fendo.2019.00676)

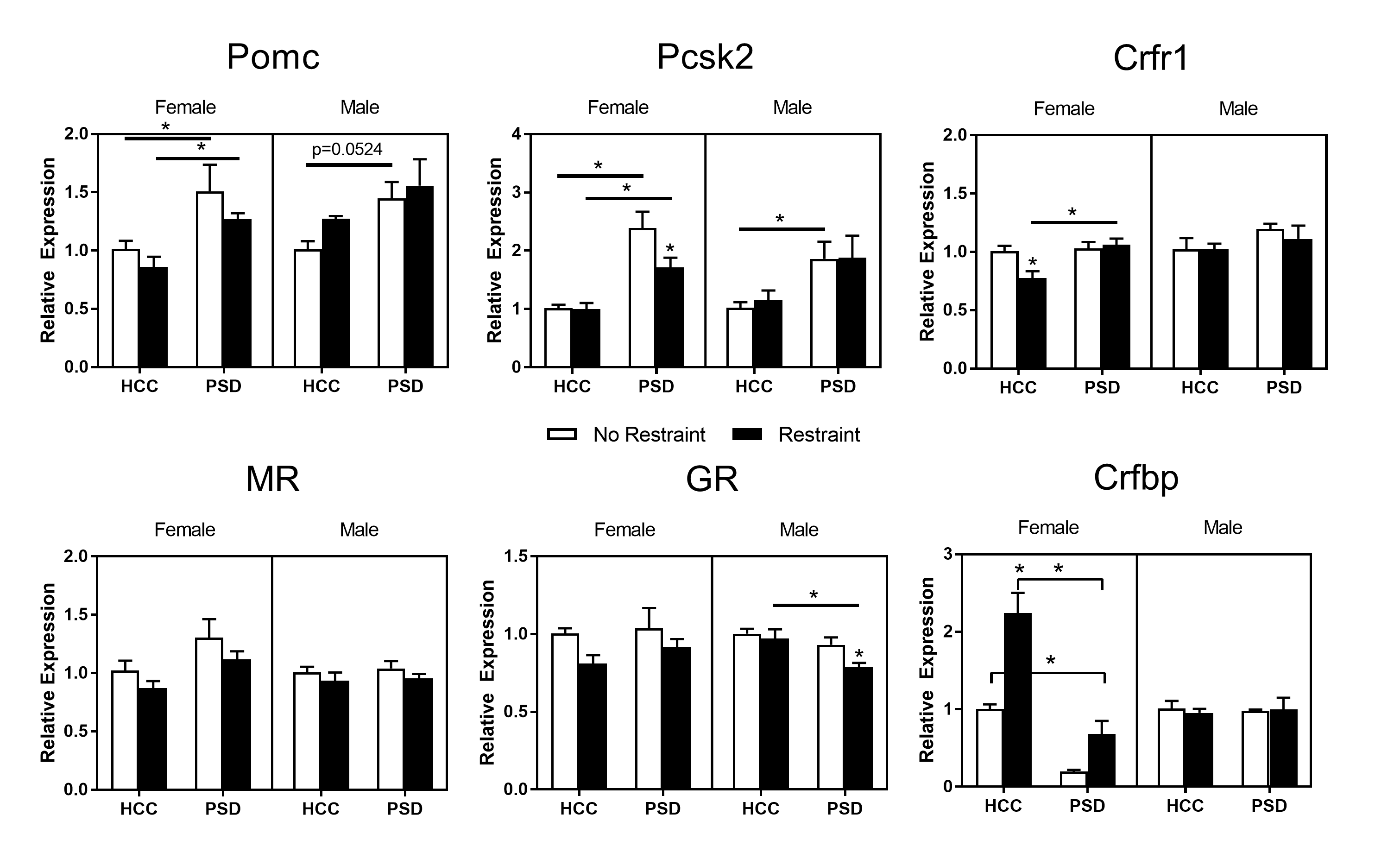

Supplement: Supplementary Figure S1 — Validation of gene expression in the pituitary using qRT-PCR. HCC, home cage controls; PSD, paradoxical sleep deprivation; CORT, corticosterone. *p < 0.05. Data are expressed as mean percentage ± SEM. [file Image_1.TIF]

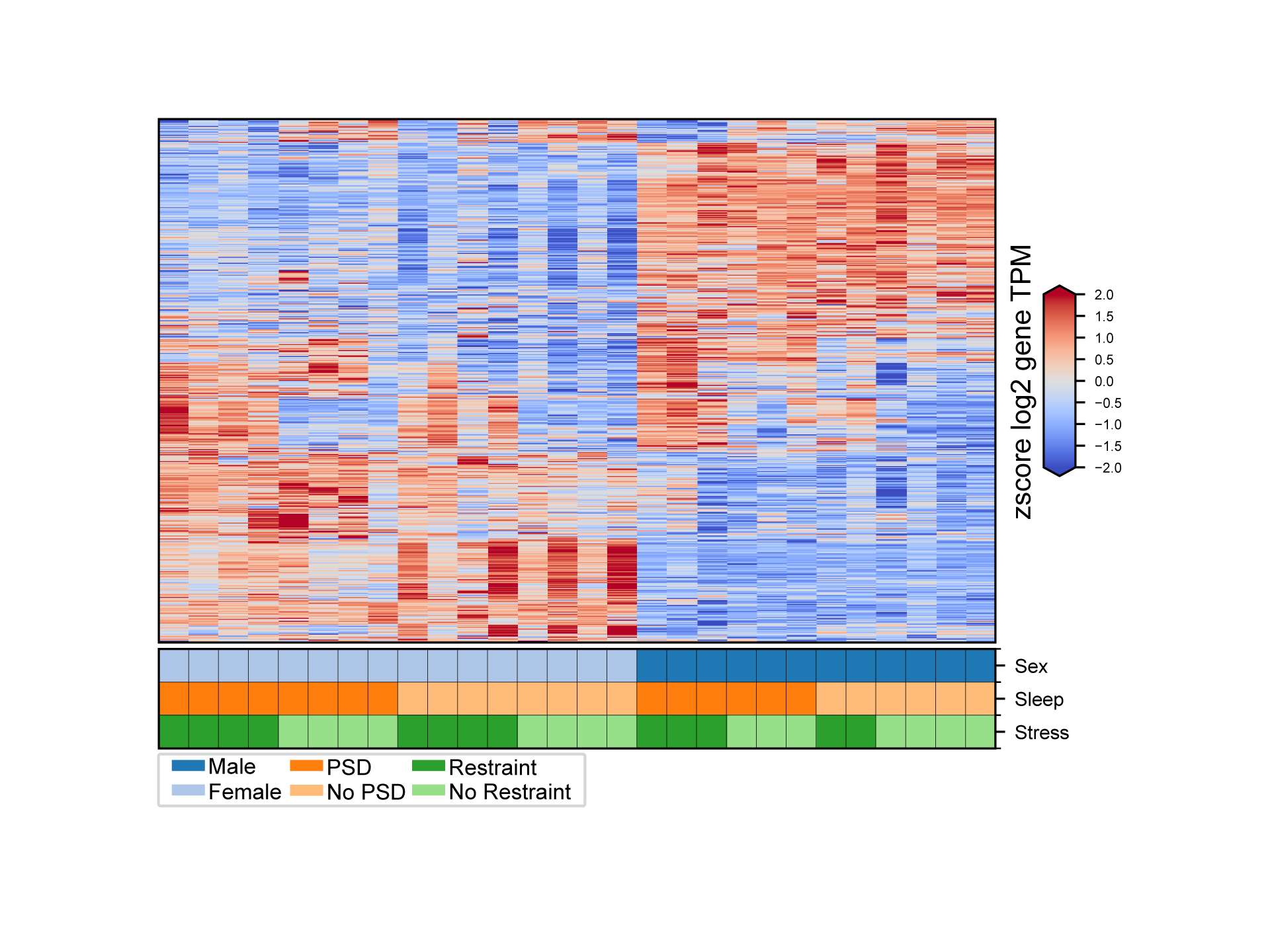

Supplement: Supplementary Figure S2 — Sex-dependent transcriptional changes along with PSD and Restraint induced alterations. Expression levels for genes differentially expressed between male and female mice (1,317 genes). Genes altered following PSD alone (400 genes), restraint alone (184 genes), and combination PSD + restraint (733 genes) are also displayed. Heatmap values are z-scored log2 TPM gene expression levels. The lower panel indicates mouse sex (top row, male or female), PSD (middle row, PSD or no PSD), and restraint (bottom row, restraint or no restraint). [file Image_2.TIF]
