# Supplementary material for: Sleep Deprivation Alters the Pituitary Stress Transcriptome in Male and Female Mice
Source: Front Endocrinol (Lausanne). 2019 Oct 9;10:676. doi: 10.3389/fendo.2019.00676 (PMC6794367; doi:10.3389/fendo.2019.00676)
Supplement: Supplementary Table 1 — Primer sequences used for real-time qPCR. [file Table_1.pdf]

Table s1: Primer sequences used for real-time qPCR

Gene	Forward primer (5′–3′)	Reverse primer (5′–3′)	Acc number
**Pomc**	CGACAGGCAGGAGACTGAAC	AGATGCGCAGAGAAACGAGG	NM_007618.3
**Pcsk2**	AAGAAGACGCAGCCTACACC	TTGCCCAGTGTTGAACAGGT	NM_008792.4
**Crfr1**	AGGATCAGCAGTGTGAGAGC	AGCCATTGTTTGTGGTGTTGTAG	NM_007762.5
**Crhbp**	CAGTTAAAGAAACCTGCGGCT	TTTCCCACTGGAGACCATGC	NM_198408.3
**Nr3c1 (GR)**	AGTGGAAGGACAGCACAATTA	TACTCCGTTTGCTTGGGGAC	NM_008173.3
**Nr3c2 (MR)**	GATGGGTACCCGGTCCTAGA	GTTGTGTTGTCCTTCCACGG	NM_001083906.1
**TBP**	CCTATCACTCCTGCCACACC	ATGACTGCAGCAAATCGCTTG	NM_013684.3
