# Supplementary material for: Sleep Deprivation Alters the Pituitary Stress Transcriptome in Male and Female Mice
Source: Front Endocrinol (Lausanne). 2019 Oct 9;10:676. doi: 10.3389/fendo.2019.00676 (PMC6794367; doi:10.3389/fendo.2019.00676)
Supplement: Supplementary Table 2 — Female: PSD Up. [file Table_2.pdf]

Female: PSD Up

GO_term	name	ontology	pvalue	FDR
GO:0007610	behavior	biological_process	9.84E-08	0.00011
GO:0043269	regulation of ion transport	biological_process	4.10E-07	0.00023
GO:0050801	ion homeostasis	biological_process	5.18E-07	0.000247
GO:0098660	inorganic ion transmembrane transport	biological_process	5.51E-07	0.000247
GO:0055080	cation homeostasis	biological_process	6.74E-07	0.000275
GO:0030003	cellular cation homeostasis	biological_process	9.35E-07	0.000321
GO:0098771	inorganic ion homeostasis	biological_process	9.56E-07	0.000321
GO:0006873	cellular ion homeostasis	biological_process	1.20E-06	0.00036
GO:0043270	positive regulation of ion transport	biological_process	1.28E-06	0.00036
GO:0006875	cellular metal ion homeostasis	biological_process	1.58E-06	0.000387
GO:0030001	metal ion transport	biological_process	1.72E-06	0.000387
GO:0098662	inorganic cation transmembrane transport	biological_process	2.36E-06	0.000504
GO:0023052	signaling	biological_process	2.70E-06	0.000551
GO:0006812	cation transport	biological_process	2.97E-06	0.00058
GO:0055065	metal ion homeostasis	biological_process	3.75E-06	0.0007
GO:0007626	locomotory behavior	biological_process	4.74E-06	0.000787
GO:0098655	cation transmembrane transport	biological_process	5.05E-06	0.000809
GO:0051952	regulation of amine transport	biological_process	6.30E-06	0.000914
GO:0030534	adult behavior	biological_process	6.32E-06	0.000914
GO:0007417	central nervous system development	biological_process	6.74E-06	0.000946
GO:0048878	chemical homeostasis	biological_process	7.84E-06	0.00107
GO:0034220	ion transmembrane transport	biological_process	1.08E-05	0.00141
GO:0055082	cellular chemical homeostasis	biological_process	1.13E-05	0.00141
GO:0042063	gliogenesis	biological_process	1.36E-05	0.00163
GO:0030182	neuron differentiation	biological_process	1.65E-05	0.0019
GO:0044070	regulation of anion transport	biological_process	1.75E-05	0.00197
GO:0043408	regulation of MAPK cascade	biological_process	2.19E-05	0.0022
GO:0048666	neuron development	biological_process	2.20E-05	0.0022
GO:1903793	positive regulation of anion transport	biological_process	2.89E-05	0.0027
GO:0002028	regulation of sodium ion transport	biological_process	3.19E-05	0.00275
GO:0072503	cellular divalent inorganic cation homeostasis	biological_process	3.33E-05	0.00282
GO:0001936	regulation of endothelial cell proliferation	biological_process	4.61E-05	0.00376
GO:0050804	modulation of chemical synaptic transmission	biological_process	5.15E-05	0.00412
GO:0007267	cell-cell signaling	biological_process	5.67E-05	0.00438
GO:0055085	transmembrane transport	biological_process	5.98E-05	0.00455
GO:0072507	divalent inorganic cation homeostasis	biological_process	6.41E-05	0.00467
GO:0032890	regulation of organic acid transport	biological_process	6.82E-05	0.00471
GO:0032892	positive regulation of organic acid transport	biological_process	7.44E-05	0.00506
GO:1901628	positive regulation of postsynaptic membrane organization	biological_process	7.89E-05	0.00528
GO:0051955	regulation of amino acid transport	biological_process	8.87E-05	0.00585
GO:0043410	positive regulation of MAPK cascade	biological_process	9.56E-05	0.00622

Female: PSD Up

GO:0042391	regulation of membrane potential	biological_process	0.000101	0.00641
GO:0051480	regulation of cytosolic calcium ion concentration	biological_process	0.000124	0.00772
GO:0044057	regulation of system process	biological_process	0.000144	0.0088
GO:0008016	regulation of heart contraction	biological_process	0.000145	0.0088
GO:0060688	regulation of morphogenesis of a branching structure	biological_process	0.000158	0.00922
GO:0019725	cellular homeostasis	biological_process	0.00016	0.00922
GO:0051957	positive regulation of amino acid transport	biological_process	0.000166	0.00944
GO:0008015	blood circulation	biological_process	0.000199	0.0106
GO:0009306	protein secretion	biological_process	0.000206	0.0107
GO:0010959	regulation of metal ion transport	biological_process	0.000214	0.011
GO:0045161	neuronal ion channel clustering	biological_process	0.000216	0.011
GO:0010001	glial cell differentiation	biological_process	0.000222	0.0112
GO:0051954	positive regulation of amine transport	biological_process	0.000224	0.0112
GO:0051093	negative regulation of developmental process	biological_process	0.000229	0.0113
GO:0003013	circulatory system process	biological_process	0.000233	0.0113
GO:0071805	potassium ion transmembrane transport	biological_process	0.000233	0.0113
GO:0006874	cellular calcium ion homeostasis	biological_process	0.000251	0.0118
GO:0006813	potassium ion transport	biological_process	0.000252	0.0118
GO:0071804	cellular potassium ion transport	biological_process	0.000253	0.0118
GO:0010963	regulation of L-arginine import	biological_process	0.000257	0.0118
GO:0034230	enkephalin processing	biological_process	0.000257	0.0118
GO:0008344	adult locomotory behavior	biological_process	0.000274	0.0124
GO:0008284	positive regulation of cell proliferation	biological_process	0.00028	0.0126
GO:0019934	cGMP-mediated signaling	biological_process	0.00032	0.0139
GO:1903909	regulation of receptor clustering	biological_process	0.00032	0.0139
GO:0051961	negative regulation of nervous system development	biological_process	0.000338	0.0144
GO:0055074	calcium ion homeostasis	biological_process	0.000351	0.0147
GO:0002790	peptide secretion	biological_process	0.000445	0.018
GO:0034219	carbohydrate transmembrane transport	biological_process	0.000451	0.0181
GO:1905330	regulation of morphogenesis of an epithelium	biological_process	0.000495	0.0197
GO:0007612	learning	biological_process	0.000502	0.0197
GO:0000041	transition metal ion transport	biological_process	0.000509	0.0199
GO:0048812	neuron projection morphogenesis	biological_process	0.000567	0.0214
GO:0007263	nitric oxide mediated signal transduction	biological_process	0.000613	0.0225
GO:0033604	negative regulation of catecholamine secretion	biological_process	0.000613	0.0225
GO:0050768	negative regulation of neurogenesis	biological_process	0.000618	0.0225
GO:0042551	neuron maturation	biological_process	0.000629	0.0228
GO:0050678	regulation of epithelial cell proliferation	biological_process	0.00064	0.023
GO:0016525	negative regulation of angiogenesis	biological_process	0.000654	0.0232
GO:0120039	plasma membrane bounded cell projection morphogenesis	biological_process	0.000656	0.0232

Female: PSD Up

GO:0048858	cell projection morphogenesis	biological_process	0.000756	0.0255
GO:0010958	regulation of amino acid import	biological_process	0.000762	0.0255
GO:0030070	insulin processing	biological_process	0.000762	0.0255
GO:0071673	positive regulation of smooth muscle cell chemotaxis	biological_process	0.000762	0.0255
GO:1901629	regulation of presynaptic membrane organization	biological_process	0.000762	0.0255
GO:2000181	negative regulation of blood vessel morphogenesis	biological_process	0.000767	0.0255
GO:0008217	regulation of blood pressure	biological_process	0.000787	0.026
GO:0000902	cell morphogenesis	biological_process	0.000807	0.0261
GO:0006883	cellular sodium ion homeostasis	biological_process	0.000807	0.0261
GO:1901626	regulation of postsynaptic membrane organization	biological_process	0.000807	0.0261
GO:0019935	cyclic-nucleotide-mediated signaling	biological_process	0.00082	0.0263
GO:0031175	neuron projection development	biological_process	0.000825	0.0263
GO:0006911	phagocytosis, engulfment	biological_process	0.00085	0.0267
GO:1903522	regulation of blood circulation	biological_process	0.000861	0.0268
GO:0070838	divalent metal ion transport	biological_process	0.000932	0.0285
GO:0072511	divalent inorganic cation transport	biological_process	0.000971	0.0289
GO:0001937	negative regulation of endothelial cell proliferation	biological_process	0.00098	0.0289
GO:0007618	mating	biological_process	0.00098	0.0289
GO:0048709	oligodendrocyte differentiation	biological_process	0.000998	0.0291
GO:0035023	regulation of Rho protein signal transduction	biological_process	0.00101	0.0292
GO:0050679	positive regulation of epithelial cell proliferation	biological_process	0.00108	0.0306
GO:1902533	positive regulation of intracellular signal transduction	biological_process	0.00109	0.0308
GO:0022603	regulation of anatomical structure morphogenesis	biological_process	0.00111	0.0309
GO:0046903	secretion	biological_process	0.00111	0.0309
GO:1901343	negative regulation of vasculature development	biological_process	0.00112	0.0309
GO:0050663	cytokine secretion	biological_process	0.00112	0.0309
GO:0061564	axon development	biological_process	0.00118	0.0324
GO:0008285	negative regulation of cell proliferation	biological_process	0.00122	0.0332
GO:0007205	protein kinase C-activating G-protein coupled receptor signaling pathway	biological_process	0.0013	0.0338
GO:0007638	mechanosensory behavior	biological_process	0.0013	0.0338
GO:0010544	negative regulation of platelet activation	biological_process	0.0013	0.0338
GO:0031643	positive regulation of myelination	biological_process	0.0013	0.0338
GO:0034312	diol biosynthetic process	biological_process	0.0013	0.0338
GO:0042559	pteridine-containing compound biosynthetic process	biological_process	0.0013	0.0338
GO:0032990	cell part morphogenesis	biological_process	0.00137	0.0347
GO:0019233	sensory perception of pain	biological_process	0.00144	0.0362
GO:0007411	axon guidance	biological_process	0.00144	0.0362

Female: PSD Up

GO:0034380	high-density lipoprotein particle assembly	biological_process	0.00151	0.0364
GO:1903275	positive regulation of sodium ion export	biological_process	0.00151	0.0364
GO:1903278	positive regulation of sodium ion export from cell	biological_process	0.00151	0.0364
GO:1904395	positive regulation of skeletal muscle acetylcholine-gated channel clustering	biological_process	0.00151	0.0364
GO:0097485	neuron projection guidance	biological_process	0.00151	0.0364
GO:0007611	learning or memory	biological_process	0.00154	0.0368
GO:0010721	negative regulation of cell development	biological_process	0.00157	0.0371
GO:0035150	regulation of tube size	biological_process	0.0016	0.0371
GO:0050880	regulation of blood vessel size	biological_process	0.0016	0.0371
GO:0061000	negative regulation of dendritic spine development	biological_process	0.00161	0.0371
GO:0006814	sodium ion transport	biological_process	0.00168	0.0386
GO:1901342	regulation of vasculature development	biological_process	0.00172	0.039
GO:0015672	monovalent inorganic cation transport	biological_process	0.00179	0.0403
GO:0001938	positive regulation of endothelial cell proliferation	biological_process	0.00185	0.0413
GO:0034103	regulation of tissue remodeling	biological_process	0.00185	0.0413
GO:0042327	positive regulation of phosphorylation	biological_process	0.00191	0.0424
GO:0010039	response to iron ion	biological_process	0.00196	0.0431
GO:0035094	response to nicotine	biological_process	0.00196	0.0431
GO:0007409	axonogenesis	biological_process	0.002	0.0437
GO:0007631	feeding behavior	biological_process	0.00201	0.0437
GO:0035725	sodium ion transmembrane transport	biological_process	0.00206	0.0446
GO:2000027	regulation of organ morphogenesis	biological_process	0.00218	0.0466
GO:0001934	positive regulation of protein phosphorylation	biological_process	0.00218	0.0466
GO:0050680	negative regulation of epithelial cell proliferation	biological_process	0.00219	0.0466
GO:0050433	regulation of catecholamine secretion	biological_process	0.00229	0.0481
GO:0099024	plasma membrane invagination	biological_process	0.00229	0.0481
GO:0030799	regulation of cyclic nucleotide metabolic process	biological_process	0.0023	0.0481
GO:0034311	diol metabolic process	biological_process	0.00235	0.0487
GO:0002035	brain renin-angiotensin system	biological_process	0.00249	0.0487
GO:0015670	carbon dioxide transport	biological_process	0.00249	0.0487
GO:0032489	regulation of Cdc42 protein signal transduction	biological_process	0.00249	0.0487
GO:0071671	regulation of smooth muscle cell chemotaxis	biological_process	0.00249	0.0487
GO:1903273	regulation of sodium ion export	biological_process	0.00249	0.0487
GO:1903276	regulation of sodium ion export from cell	biological_process	0.00249	0.0487
GO:1904393	regulation of skeletal muscle acetylcholine-gated channel clustering	biological_process	0.00249	0.0487
GO:1904398	positive regulation of neuromuscular junction development	biological_process	0.00249	0.0487
GO:0031644	regulation of neurological system process	biological_process	0.00253	0.0489
GO:0001505	regulation of neurotransmitter levels	biological_process	0.00254	0.0489

Female: PSD Up

GO:0050866	negative regulation of cell activation	biological_process	0.00254	0.0489
GO:0010765	positive regulation of sodium ion transport	biological_process	0.00255	0.0489
GO:0055078	sodium ion homeostasis	biological_process	0.00279	0.053
GO:0045765	regulation of angiogenesis	biological_process	0.00283	0.0537
GO:0002027	regulation of heart rate	biological_process	0.00293	0.055
GO:0045596	negative regulation of cell differentiation	biological_process	0.00294	0.055
GO:0032989	cellular component morphogenesis	biological_process	0.00301	0.0561
GO:0050905	neuromuscular process	biological_process	0.00308	0.0569
GO:0051046	regulation of secretion	biological_process	0.00312	0.0574
GO:0007162	negative regulation of cell adhesion	biological_process	0.00316	0.0576
GO:0099537	trans-synaptic signaling	biological_process	0.00317	0.0577
GO:0001990	regulation of systemic arterial blood pressure by hormone	biological_process	0.00327	0.0583
GO:0051953	negative regulation of amine transport	biological_process	0.00327	0.0583
GO:0099536	synaptic signaling	biological_process	0.00337	0.0594
GO:0009798	axis specification	biological_process	0.00337	0.0594
GO:0031099	regeneration	biological_process	0.00337	0.0594
GO:0050890	cognition	biological_process	0.0034	0.0595
GO:0001816	cytokine production	biological_process	0.00344	0.0598
GO:0045995	regulation of embryonic development	biological_process	0.00344	0.0598
GO:0048167	regulation of synaptic plasticity	biological_process	0.00351	0.0598
GO:1903530	regulation of secretion by cell	biological_process	0.00353	0.0598
GO:0055067	monovalent inorganic cation homeostasis	biological_process	0.00364	0.0598
GO:0006707	cholesterol catabolic process	biological_process	0.00369	0.0598
GO:0010624	regulation of Schwann cell proliferation	biological_process	0.00369	0.0598
GO:0010873	positive regulation of cholesterol esterification	biological_process	0.00369	0.0598
GO:0016127	sterol catabolic process	biological_process	0.00369	0.0598
GO:0032525	somite rostral/caudal axis specification	biological_process	0.00369	0.0598
GO:0033700	phospholipid efflux	biological_process	0.00369	0.0598
GO:0035428	hexose transmembrane transport	biological_process	0.00369	0.0598
GO:0036376	sodium ion export from cell	biological_process	0.00369	0.0598
GO:0046654	tetrahydrofolate biosynthetic process	biological_process	0.00369	0.0598
GO:1903911	positive regulation of receptor clustering	biological_process	0.00369	0.0598
GO:1903961	positive regulation of anion transmembrane transport	biological_process	0.00369	0.0598
GO:1905906	regulation of amyloid fibril formation	biological_process	0.00369	0.0598
GO:1905950	monosaccharide transmembrane transport	biological_process	0.00369	0.0598
GO:0046850	regulation of bone remodeling	biological_process	0.00376	0.0604
GO:0061045	negative regulation of wound healing	biological_process	0.00376	0.0604
GO:0031102	neuron projection regeneration	biological_process	0.00381	0.0604
GO:0042417	dopamine metabolic process	biological_process	0.00381	0.0604
GO:0042755	eating behavior	biological_process	0.00381	0.0604
GO:0046916	cellular transition metal ion homeostasis	biological_process	0.00386	0.061
GO:0070372	regulation of ERK1 and ERK2 cascade	biological_process	0.00389	0.0613

Female: PSD Up

GO:0008544	epidermis development	biological_process	0.00402	0.0626
GO:0055123	digestive system development	biological_process	0.00404	0.0626
GO:0070374	positive regulation of ERK1 and ERK2 cascade	biological_process	0.00405	0.0626
GO:0032940	secretion by cell	biological_process	0.0041	0.0629
GO:0010324	membrane invagination	biological_process	0.00412	0.0629
GO:0019226	transmission of nerve impulse	biological_process	0.00412	0.0629
GO:0098657	import into cell	biological_process	0.00412	0.0629
GO:0003018	vascular process in circulatory system	biological_process	0.00423	0.0643
GO:0055076	transition metal ion homeostasis	biological_process	0.00426	0.0646
GO:0030195	negative regulation of blood coagulation	biological_process	0.00439	0.0654
GO:0045992	negative regulation of embryonic development	biological_process	0.00439	0.0654
GO:1900047	negative regulation of hemostasis	biological_process	0.00439	0.0654
GO:0035296	regulation of tube diameter	biological_process	0.00496	0.0708
GO:0070588	calcium ion transmembrane transport	biological_process	0.00496	0.0708
GO:0097746	regulation of blood vessel diameter	biological_process	0.00496	0.0708
GO:0022404	molting cycle process	biological_process	0.00499	0.0708
GO:0022405	hair cycle process	biological_process	0.00499	0.0708
GO:0050819	negative regulation of coagulation	biological_process	0.00502	0.0708
GO:0002016	regulation of blood volume by renin-angiotensin	biological_process	0.00511	0.0708
GO:0006729	tetrahydrobiopterin biosynthetic process	biological_process	0.00511	0.0708
GO:0042297	vocal learning	biological_process	0.00511	0.0708
GO:0043691	reverse cholesterol transport	biological_process	0.00511	0.0708
GO:0045916	negative regulation of complement activation	biological_process	0.00511	0.0708
GO:0046146	tetrahydrobiopterin metabolic process	biological_process	0.00511	0.0708
GO:0071436	sodium ion export	biological_process	0.00511	0.0708
GO:0098596	imitative learning	biological_process	0.00511	0.0708
GO:2000258	negative regulation of protein activation cascade	biological_process	0.00511	0.0708
GO:0120036	plasma membrane bounded cell projection organization	biological_process	0.00515	0.0711
GO:0009266	response to temperature stimulus	biological_process	0.00521	0.0717
GO:0006820	anion transport	biological_process	0.00539	0.0738
GO:0014002	astrocyte development	biological_process	0.00571	0.0773
GO:0031646	positive regulation of neurological system process	biological_process	0.00571	0.0773
GO:0090288	negative regulation of cellular response to growth factor stimulus	biological_process	0.00573	0.0773
GO:0061024	membrane organization	biological_process	0.00574	0.0773
GO:0015711	organic anion transport	biological_process	0.00577	0.0775
GO:0009636	response to toxic substance	biological_process	0.00598	0.0791
GO:0021782	glial cell development	biological_process	0.00598	0.0791
GO:0042303	molting cycle	biological_process	0.00598	0.0791
GO:0042633	hair cycle	biological_process	0.00598	0.0791
GO:0009628	response to abiotic stimulus	biological_process	0.00619	0.0815

Female: PSD Up

GO:0003044	regulation of systemic arterial blood pressure mediated by a chemical signal	biological_process	0.00645	0.0843
GO:0010817	regulation of hormone levels	biological_process	0.00656	0.0843
GO:0032101	regulation of response to external stimulus	biological_process	0.00672	0.0843
GO:0006563	L-serine metabolic process	biological_process	0.00674	0.0843
GO:0007202	activation of phospholipase C activity	biological_process	0.00674	0.0843
GO:0010872	regulation of cholesterol esterification	biological_process	0.00674	0.0843
GO:0014052	regulation of gamma-aminobutyric acid secretion	biological_process	0.00674	0.0843
GO:0015669	gas transport	biological_process	0.00674	0.0843
GO:0015740	C4-dicarboxylate transport	biological_process	0.00674	0.0843
GO:0019755	one-carbon compound transport	biological_process	0.00674	0.0843
GO:0030007	cellular potassium ion homeostasis	biological_process	0.00674	0.0843
GO:0042416	dopamine biosynthetic process	biological_process	0.00674	0.0843
GO:1900221	regulation of amyloid-beta clearance	biological_process	0.00674	0.0843
GO:1902430	negative regulation of amyloid-beta formation	biological_process	0.00674	0.0843
GO:0010977	negative regulation of neuron projection development	biological_process	0.00691	0.0861
GO:0030004	cellular monovalent inorganic cation homeostasis	biological_process	0.0071	0.0882
GO:0045823	positive regulation of heart contraction	biological_process	0.00724	0.0892
GO:0070207	protein homotrimerization	biological_process	0.00724	0.0892
GO:1902305	regulation of sodium ion transmembrane transport	biological_process	0.00725	0.0892
GO:0009612	response to mechanical stimulus	biological_process	0.00756	0.0925
GO:0018958	phenol-containing compound metabolic process	biological_process	0.0078	0.0946
GO:0010038	response to metal ion	biological_process	0.00786	0.0951
GO:1903531	negative regulation of secretion by cell	biological_process	0.00803	0.096
GO:0007268	chemical synaptic transmission	biological_process	0.00808	0.096
GO:0098916	anterograde trans-synaptic signaling	biological_process	0.00808	0.096
GO:0010543	regulation of platelet activation	biological_process	0.00809	0.096
GO:0042311	vasodilation	biological_process	0.00809	0.096
GO:0048546	digestive tract morphogenesis	biological_process	0.00809	0.096
GO:0050730	regulation of peptidyl-tyrosine phosphorylation	biological_process	0.00839	0.0993
GO:0009396	folic acid-containing compound biosynthetic process	biological_process	0.00858	0.0997
GO:0042428	serotonin metabolic process	biological_process	0.00858	0.0997
GO:0071281	cellular response to iron ion	biological_process	0.00858	0.0997
GO:0098597	observational learning	biological_process	0.00858	0.0997
GO:0098598	learned vocalization behavior or vocal learning	biological_process	0.00858	0.0997
GO:1904396	regulation of neuromuscular junction development	biological_process	0.00858	0.0997
GO:0030425	dendrite	cellular_component	3.74E-07	0.00023
GO:0044456	synapse part	cellular_component	1.00E-06	0.000321

Female: PSD Up

GO:0036477	somatodendritic compartment	cellular_component	1.64E-06	0.000387
GO:0072562	blood microparticle	cellular_component	4.31E-06	0.000756
GO:0045202	synapse	cellular_component	4.38E-06	0.000756
GO:0044297	cell body	cellular_component	5.48E-06	0.000847
GO:0043025	neuronal cell body	cellular_component	2.21E-05	0.0022
GO:0098793	presynapse	cellular_component	2.33E-05	0.00228
GO:0098590	plasma membrane region	cellular_component	3.10E-05	0.00275
GO:0005901	caveola	cellular_component	3.19E-05	0.00275
GO:0099503	secretory vesicle	cellular_component	5.63E-05	0.00438
GO:0044304	main axon	cellular_component	6.49E-05	0.00467
GO:0044853	plasma membrane raft	cellular_component	9.82E-05	0.0063
GO:0098589	membrane region	cellular_component	0.000149	0.00891
GO:1902495	transmembrane transporter complex	cellular_component	0.000152	0.00896
GO:0030659	cytoplasmic vesicle membrane	cellular_component	0.000193	0.0106
GO:1990351	transporter complex	cellular_component	0.000197	0.0106
GO:0045121	membrane raft	cellular_component	0.000199	0.0106
GO:0098857	membrane microdomain	cellular_component	0.000199	0.0106
GO:0009986	cell surface	cellular_component	0.000201	0.0106
GO:0097060	synaptic membrane	cellular_component	0.000292	0.013
GO:0030667	secretory granule membrane	cellular_component	0.000324	0.014
GO:0048471	perinuclear region of cytoplasm	cellular_component	0.000339	0.0144
GO:0031012	extracellular matrix	cellular_component	0.000369	0.0153
GO:0012506	vesicle membrane	cellular_component	0.000528	0.0204
GO:0034364	high-density lipoprotein particle	cellular_component	0.000613	0.0225
GO:0008021	synaptic vesicle	cellular_component	0.00068	0.0238
GO:0034705	potassium channel complex	cellular_component	0.000709	0.0247
GO:0032809	neuronal cell body membrane	cellular_component	0.00085	0.0267
GO:0042734	presynaptic membrane	cellular_component	0.000906	0.0279
GO:0030141	secretory granule	cellular_component	0.000907	0.0279
GO:0044298	cell body membrane	cellular_component	0.00098	0.0289
GO:0034702	ion channel complex	cellular_component	0.00105	0.0301
GO:0043204	perikaryon	cellular_component	0.00128	0.0338
GO:0033267	axon part	cellular_component	0.00133	0.0342
GO:0070382	exocytic vesicle	cellular_component	0.00134	0.0342
GO:0002080	acrosomal membrane	cellular_component	0.00161	0.0371
GO:0034703	cation channel complex	cellular_component	0.00228	0.0481
GO:0031232	extrinsic component of external side of plasma membrane	cellular_component	0.00249	0.0487
GO:0044433	cytoplasmic vesicle part	cellular_component	0.0025	0.0487
GO:0008076	voltage-gated potassium channel complex	cellular_component	0.00293	0.055
GO:0044291	cell-cell contact zone	cellular_component	0.00315	0.0576
GO:0034358	plasma lipoprotein particle	cellular_component	0.00327	0.0583
GO:1990777	lipoprotein particle	cellular_component	0.00327	0.0583
GO:0005890	sodium:potassium-exchanging ATPase complex	cellular_component	0.00369	0.0598

Female: PSD Up

GO:0043203	axon hillock	cellular_component	0.00369	0.0598
GO:0032994	protein-lipid complex	cellular_component	0.00439	0.0654
GO:0032589	neuron projection membrane	cellular_component	0.00449	0.0668
GO:0030133	transport vesicle	cellular_component	0.00476	0.0702
GO:0090533	cation-transporting ATPase complex	cellular_component	0.00674	0.0843
GO:0098533	ATPase dependent transmembrane transport complex	cellular_component	0.00674	0.0843
GO:0098794	postsynapse	cellular_component	0.00716	0.0888
GO:0098552	side of membrane	cellular_component	0.00771	0.094
GO:0014704	intercalated disc	cellular_component	0.0078	0.0946
GO:0046873	metal ion transmembrane transporter activity	molecular_function	6.41E-09	2.88E-05
GO:0022891	substrate-specific transmembrane transporter activity	molecular_function	4.76E-08	0.000107
GO:0008324	cation transmembrane transporter activity	molecular_function	9.61E-08	0.00011
GO:0015075	ion transmembrane transporter activity	molecular_function	1.22E-07	0.00011
GO:0022890	inorganic cation transmembrane transporter activity	molecular_function	2.94E-07	0.00022
GO:0015081	sodium ion transmembrane transporter activity	molecular_function	1.59E-06	0.000387
GO:0030545	receptor regulator activity	molecular_function	1.12E-05	0.00141
GO:0048018	receptor ligand activity	molecular_function	1.38E-05	0.00163
GO:0015077	monovalent inorganic cation transmembrane transporter activity	molecular_function	1.95E-05	0.00213
GO:0005261	cation channel activity	molecular_function	2.13E-05	0.0022
GO:0005216	ion channel activity	molecular_function	2.57E-05	0.00245
GO:0022838	substrate-specific channel activity	molecular_function	3.05E-05	0.00275
GO:0015079	potassium ion transmembrane transporter activity	molecular_function	4.46E-05	0.00371
GO:0017147	Wnt-protein binding	molecular_function	6.19E-05	0.00463
GO:0015267	channel activity	molecular_function	6.66E-05	0.00467
GO:0022803	passive transmembrane transporter activity	molecular_function	6.66E-05	0.00467
GO:0004867	serine-type endopeptidase inhibitor activity	molecular_function	0.000377	0.0155
GO:0022804	active transmembrane transporter activity	molecular_function	0.000379	0.0155
GO:0001664	G-protein coupled receptor binding	molecular_function	0.000536	0.0205
GO:0015085	calcium ion transmembrane transporter activity	molecular_function	0.000568	0.0214
GO:0072509	divalent inorganic cation transmembrane transporter activity	molecular_function	0.000971	0.0289
GO:0005179	hormone activity	molecular_function	0.000998	0.0291
GO:0005262	calcium channel activity	molecular_function	0.00104	0.0299
GO:0005184	neuropeptide hormone activity	molecular_function	0.0013	0.0338
GO:0005509	calcium ion binding	molecular_function	0.00134	0.0342
GO:0004930	G-protein coupled receptor activity	molecular_function	0.00146	0.0364
GO:0005250	A-type (transient outward) potassium channel activity	molecular_function	0.00151	0.0364
GO:0030165	PDZ domain binding	molecular_function	0.00151	0.0364

Female: PSD Up

GO:0097110	scaffold protein binding	molecular_function	0.00157	0.0371
GO:0005343	organic acid:sodium symporter activity	molecular_function	0.00161	0.0371
GO:0022836	gated channel activity	molecular_function	0.00172	0.039
GO:0004866	endopeptidase inhibitor activity	molecular_function	0.00217	0.0466
GO:0030414	peptidase inhibitor activity	molecular_function	0.00245	0.0487
GO:0004865	protein serine/threonine phosphatase inhibitor activity	molecular_function	0.00249	0.0487
GO:0008199	ferric iron binding	molecular_function	0.00249	0.0487
GO:0031749	D2 dopamine receptor binding	molecular_function	0.00249	0.0487
GO:0022843	voltage-gated cation channel activity	molecular_function	0.0026	0.0496
GO:0061135	endopeptidase regulator activity	molecular_function	0.00308	0.0569
GO:0050780	dopamine receptor binding	molecular_function	0.00327	0.0583
GO:0019238	cyclohydrolase activity	molecular_function	0.00369	0.0598
GO:0042813	Wnt-activated receptor activity	molecular_function	0.00381	0.0604
GO:0005244	voltage-gated ion channel activity	molecular_function	0.00405	0.0626
GO:0022832	voltage-gated channel activity	molecular_function	0.00405	0.0626
GO:1905030	voltage-gated ion channel activity involved in regulation of postsynaptic membrane potential	molecular_function	0.00439	0.0654
GO:0005539	glycosaminoglycan binding	molecular_function	0.00461	0.0682
GO:0015179	L-amino acid transmembrane transporter activity	molecular_function	0.00489	0.0708
GO:0005391	sodium:potassium-exchanging ATPase activity	molecular_function	0.00511	0.0708
GO:0008556	potassium-transporting ATPase activity	molecular_function	0.00511	0.0708
GO:0031995	insulin-like growth factor II binding	molecular_function	0.00511	0.0708
GO:0048406	nerve growth factor binding	molecular_function	0.00511	0.0708
GO:0001540	amyloid-beta binding	molecular_function	0.00532	0.073
GO:0015370	solute:sodium symporter activity	molecular_function	0.00571	0.0773
GO:0008083	growth factor activity	molecular_function	0.00598	0.0791
GO:0016810	hydrolase activity, acting on carbon-nitrogen (but not peptide) bonds	molecular_function	0.00601	0.0794
GO:0004089	carbonate dehydratase activity	molecular_function	0.00674	0.0843
GO:0005283	sodium:amino acid symporter activity	molecular_function	0.00674	0.0843
GO:0015291	secondary active transmembrane transporter activity	molecular_function	0.00745	0.0914
GO:0015175	neutral amino acid transmembrane transporter activity	molecular_function	0.00809	0.096
GO:0016595	glutamate binding	molecular_function	0.00858	0.0997
