# Supplementary material for: Sleep Deprivation Alters the Pituitary Stress Transcriptome in Male and Female Mice
Source: Front Endocrinol (Lausanne). 2019 Oct 9;10:676. doi: 10.3389/fendo.2019.00676 (PMC6794367; doi:10.3389/fendo.2019.00676)
Supplement: Supplementary Table 3 — Male: PSD Up. [file Table_3.pdf]

Male: PSD Up

GO_term	name	ontology	pvalue	FDR
GO:0046653	tetrahydrofolate metabolic process	biological_process	1.06E-05	0.00514
GO:0006760	folic acid-containing compound metabolic process	biological_process	1.92E-05	0.00514
GO:0006730	one-carbon metabolic process	biological_process	2.81E-05	0.00514
GO:0006116	NADH oxidation	biological_process	3.08E-05	0.00514
GO:0042558	pteridine-containing compound metabolic process	biological_process	3.53E-05	0.00523
GO:0009256	10-formyltetrahydrofolate metabolic process	biological_process	5.13E-05	0.00685
GO:0055114	oxidation-reduction process	biological_process	9.49E-05	0.0106
GO:0006732	coenzyme metabolic process	biological_process	0.000105	0.0107
GO:0051186	cofactor metabolic process	biological_process	0.000297	0.0283
GO:0097284	hepatocyte apoptotic process	biological_process	0.000335	0.0298
GO:0043648	dicarboxylic acid metabolic process	biological_process	0.000649	0.0542
GO:0006734	NADH metabolic process	biological_process	0.00086	0.0675
GO:0015711	organic anion transport	biological_process	0.00119	0.0788
GO:1904019	epithelial cell apoptotic process	biological_process	0.00138	0.0788
GO:0061041	regulation of wound healing	biological_process	0.00142	0.0788
GO:0006911	phagocytosis, engulfment	biological_process	0.00174	0.0788
GO:0055086	nucleobase-containing small molecule metabolic process	biological_process	0.00177	0.0788
GO:0009635	response to herbicide	biological_process	0.0023	0.0788
GO:0015794	glycerol-3-phosphate transport	biological_process	0.0023	0.0788
GO:0015942	formate metabolic process	biological_process	0.0023	0.0788
GO:0034127	regulation of MyD88-independent toll-like receptor signaling pathway	biological_process	0.0023	0.0788
GO:0097017	renal protein absorption	biological_process	0.0023	0.0788
GO:0099082	retrograde trans-synaptic signaling by neuropeptide	biological_process	0.0023	0.0788
GO:0099083	retrograde trans-synaptic signaling by neuropeptide, modulating synaptic transmission	biological_process	0.0023	0.0788
GO:1903906	regulation of plasma membrane raft polarization	biological_process	0.0023	0.0788
GO:1904674	positive regulation of somatic stem cell population maintenance	biological_process	0.0023	0.0788
GO:1904675	regulation of somatic stem cell division	biological_process	0.0023	0.0788
GO:1904677	positive regulation of somatic stem cell division	biological_process	0.0023	0.0788
GO:2000101	regulation of mammary stem cell proliferation	biological_process	0.0023	0.0788
GO:2000103	positive regulation of mammary stem cell proliferation	biological_process	0.0023	0.0788
GO:0019674	NAD metabolic process	biological_process	0.00245	0.0817
GO:0006575	cellular modified amino acid metabolic process	biological_process	0.00262	0.0819
GO:1903034	regulation of response to wounding	biological_process	0.00287	0.0819
GO:0099024	plasma membrane invagination	biological_process	0.00292	0.0819
GO:0046717	acid secretion	biological_process	0.00344	0.0819
GO:0006820	anion transport	biological_process	0.00368	0.0819
GO:0010324	membrane invagination	biological_process	0.00399	0.0819
GO:0015849	organic acid transport	biological_process	0.00446	0.0819

Male: PSD Up

GO:0046942	carboxylic acid transport	biological_process	0.00446	0.0819
GO:0006127	glycerophosphate shuttle	biological_process	0.0046	0.0819
GO:0009257	10-formyltetrahydrofolate biosynthetic process	biological_process	0.0046	0.0819
GO:0009258	10-formyltetrahydrofolate catabolic process	biological_process	0.0046	0.0819
GO:0009397	folic acid-containing compound catabolic process	biological_process	0.0046	0.0819
GO:0014891	striated muscle atrophy	biological_process	0.0046	0.0819
GO:0015688	iron chelate transport	biological_process	0.0046	0.0819
GO:0015819	lysine transport	biological_process	0.0046	0.0819
GO:0015891	siderophore transport	biological_process	0.0046	0.0819
GO:0032607	interferon-alpha production	biological_process	0.0046	0.0819
GO:0034124	regulation of MyD88-dependent toll-like receptor signaling pathway	biological_process	0.0046	0.0819
GO:0042560	pteridine-containing compound catabolic process	biological_process	0.0046	0.0819
GO:0051754	meiotic sister chromatid cohesion, centromeric	biological_process	0.0046	0.0819
GO:0061193	taste bud development	biological_process	0.0046	0.0819
GO:0099538	synaptic signaling via neuropeptide	biological_process	0.0046	0.0819
GO:0099540	trans-synaptic signaling by neuropeptide	biological_process	0.0046	0.0819
GO:0099551	trans-synaptic signaling by neuropeptide, modulating synaptic transmission	biological_process	0.0046	0.0819
GO:1903921	regulation of protein processing in phagocytic vesicle	biological_process	0.0046	0.0819
GO:1903923	positive regulation of protein processing in phagocytic vesicle	biological_process	0.0046	0.0819
GO:1904672	regulation of somatic stem cell population maintenance	biological_process	0.0046	0.0819
GO:0003012	muscle system process	biological_process	0.00481	0.0845
GO:0002190	cap-independent translational initiation	biological_process	0.00689	0.0968
GO:0002192	IRES-dependent translational initiation of linear mRNA	biological_process	0.00689	0.0968
GO:0007412	axon target recognition	biological_process	0.00689	0.0968
GO:0032608	interferon-beta production	biological_process	0.00689	0.0968
GO:0051599	response to hydrostatic pressure	biological_process	0.00689	0.0968
GO:0070601	centromeric sister chromatid cohesion	biological_process	0.00689	0.0968
GO:0098917	retrograde trans-synaptic signaling	biological_process	0.00689	0.0968
GO:0110017	cap-independent translational initiation of linear mRNA	biological_process	0.00689	0.0968
GO:1902174	positive regulation of keratinocyte apoptotic process	biological_process	0.00689	0.0968
GO:1903903	regulation of establishment of T cell polarity	biological_process	0.00689	0.0968
GO:0016646	oxidoreductase activity, acting on the CH-NH group of donors, NAD or NADP as acceptor	molecular_function	7.45E-06	0.00514
GO:0016645	oxidoreductase activity, acting on the CH-NH group of donors	molecular_function	2.49E-05	0.00514
GO:0004477	methenyltetrahydrofolate cyclohydrolase activity	molecular_function	3.08E-05	0.00514
GO:0004488	methylenetetrahydrofolate dehydrogenase (NADP+) activity	molecular_function	3.08E-05	0.00514
GO:0019238	cyclohydrolase activity	molecular_function	7.68E-05	0.00933

Male: PSD Up

GO:0016814	hydrolase activity, acting on carbon-nitrogen (but not peptide) bonds, in cyclic amidines	molecular_function	0.00126	0.0788
GO:0004648	O-phospho-L-serine:2-oxoglutarate aminotransferase activity	molecular_function	0.0023	0.0788
GO:0015169	glycerol-3-phosphate transmembrane transporter activity	molecular_function	0.0023	0.0788
GO:0031710	neuromedin B receptor binding	molecular_function	0.0023	0.0788
GO:0004867	serine-type endopeptidase inhibitor activity	molecular_function	0.00362	0.0819
GO:0004329	formate-tetrahydrofolate ligase activity	molecular_function	0.0046	0.0819
GO:0004611	phosphoenolpyruvate carboxykinase activity	molecular_function	0.0046	0.0819
GO:0004613	phosphoenolpyruvate carboxykinase (GTP) activity	molecular_function	0.0046	0.0819
GO:0005169	neurotrophin TRKB receptor binding	molecular_function	0.0046	0.0819
GO:0016155	formyltetrahydrofolate dehydrogenase activity	molecular_function	0.0046	0.0819
GO:0031705	bombesin receptor binding	molecular_function	0.0046	0.0819
GO:0045159	myosin II binding	molecular_function	0.0046	0.0819
GO:0000064	L-ornithine transmembrane transporter activity	molecular_function	0.00689	0.0968
GO:0004487	methylenetetrahydrofolate dehydrogenase (NAD+) activity	molecular_function	0.00689	0.0968
GO:0005219	ryanodine-sensitive calcium-release channel activity	molecular_function	0.00689	0.0968
GO:0015119	hexose phosphate transmembrane transporter activity	molecular_function	0.00689	0.0968
GO:0015166	polyol transmembrane transporter activity	molecular_function	0.00689	0.0968
GO:0015315	organophosphate:inorganic phosphate antiporter activity	molecular_function	0.00689	0.0968
GO:0015526	hexose-phosphate:inorganic phosphate antiporter activity	molecular_function	0.00689	0.0968
GO:0015665	alcohol transmembrane transporter activity	molecular_function	0.00689	0.0968
GO:0061513	glucose 6-phosphate:inorganic phosphate antiporter activity	molecular_function	0.00689	0.0968
