# Supplementary material for: Sleep Deprivation Alters the Pituitary Stress Transcriptome in Male and Female Mice
Source: Front Endocrinol (Lausanne). 2019 Oct 9;10:676. doi: 10.3389/fendo.2019.00676 (PMC6794367; doi:10.3389/fendo.2019.00676)
Supplement: Supplementary Table 4 — Male: PSD Down. [file Table_4.pdf]

Male: PSD Down

| GO_term | name | ontology | pvalue | FDR |
|---|---|---|---|---|
| GO:0019886 | antigen processing and presentation of exogenous peptide antigen via MHC class II | biological_process | 2.09E-08 | 6.71E-06 |
| GO:0002495 | antigen processing and presentation of peptide antigen via MHC class II | biological_process | 2.92E-08 | 6.71E-06 |
| GO:0002504 | antigen processing and presentation of peptide or polysaccharide antigen via MHC class II | biological_process | 2.92E-08 | 6.71E-06 |
| GO:0048002 | antigen processing and presentation of peptide antigen | biological_process | 3.49E-08 | 6.71E-06 |
| GO:0002478 | antigen processing and presentation of exogenous peptide antigen | biological_process | 1.40E-07 | 1.91E-05 |
| GO:0019884 | antigen processing and presentation of exogenous antigen | biological_process | 3.61E-07 | 4.02E-05 |
| GO:0019882 | antigen processing and presentation | biological_process | 3.77E-07 | 4.02E-05 |
| GO:0034341 | response to interferon-gamma | biological_process | 6.82E-06 | 0.000655 |
| GO:0002579 | positive regulation of antigen processing and presentation | biological_process | 0.00016 | 0.0139 |
| GO:0050670 | regulation of lymphocyte proliferation | biological_process | 0.000295 | 0.0211 |
| GO:0002577 | regulation of antigen processing and presentation | biological_process | 0.000312 | 0.0211 |
| GO:0032944 | regulation of mononuclear cell proliferation | biological_process | 0.000312 | 0.0211 |
| GO:0070663 | regulation of leukocyte proliferation | biological_process | 0.000359 | 0.0211 |
| GO:0022409 | positive regulation of cell-cell adhesion | biological_process | 0.000368 | 0.0211 |
| GO:0033194 | response to hydroperoxide | biological_process | 0.000374 | 0.0211 |
| GO:0050868 | negative regulation of T cell activation | biological_process | 0.00057 | 0.0304 |
| GO:0051251 | positive regulation of lymphocyte activation | biological_process | 0.000682 | 0.0345 |
| GO:1903038 | negative regulation of leukocyte cell-cell adhesion | biological_process | 0.000762 | 0.0349 |
| GO:0002696 | positive regulation of leukocyte activation | biological_process | 0.00106 | 0.0444 |
| GO:0034097 | response to cytokine | biological_process | 0.00122 | 0.049 |
| GO:0051250 | negative regulation of lymphocyte activation | biological_process | 0.0013 | 0.0494 |
| GO:0050867 | positive regulation of cell activation | biological_process | 0.00134 | 0.0494 |
| GO:0002695 | negative regulation of leukocyte activation | biological_process | 0.00182 | 0.0535 |
| GO:0030890 | positive regulation of B cell proliferation | biological_process | 0.0024 | 0.0535 |
| GO:0002343 | peripheral B cell selection | biological_process | 0.00243 | 0.0535 |
| GO:0002344 | B cell affinity maturation | biological_process | 0.00243 | 0.0535 |
| GO:0035691 | macrophage migration inhibitory factor signaling pathway | biological_process | 0.00243 | 0.0535 |
| GO:0048866 | stem cell fate specification | biological_process | 0.00243 | 0.0535 |
| GO:1904323 | regulation of inhibitory G-protein coupled receptor phosphorylation | biological_process | 0.00243 | 0.0535 |
| GO:1904325 | positive regulation of inhibitory G-protein coupled receptor phosphorylation | biological_process | 0.00243 | 0.0535 |
| GO:1990227 | paranodal junction maintenance | biological_process | 0.00243 | 0.0535 |
| GO:0022408 | negative regulation of cell-cell adhesion | biological_process | 0.00245 | 0.0535 |
| GO:0050870 | positive regulation of T cell activation | biological_process | 0.00245 | 0.0535 |
| GO:0050866 | negative regulation of cell activation | biological_process | 0.00271 | 0.0557 |
| GO:1903039 | positive regulation of leukocyte cell-cell adhesion | biological_process | 0.00285 | 0.0557 |
| GO:0071346 | cellular response to interferon-gamma | biological_process | 0.00325 | 0.0624 |

Male: PSD Down

| | | | | |
|---|---|---|---|---|
| GO:0051249 | regulation of lymphocyte activation | biological_process | 0.00363 | 0.068 |
| GO:0022407 | regulation of cell-cell adhesion | biological_process | 0.00368 | 0.068 |
| GO:0042130 | negative regulation of T cell proliferation | biological_process | 0.00403 | 0.0729 |
| GO:0045785 | positive regulation of cell adhesion | biological_process | 0.00426 | 0.0744 |
| GO:0030888 | regulation of B cell proliferation | biological_process | 0.00466 | 0.0777 |
| GO:0034769 | basement membrane disassembly | biological_process | 0.00485 | 0.0777 |
| GO:0060214 | endocardium formation | biological_process | 0.00485 | 0.0777 |
| GO:0002683 | negative regulation of immune system process | biological_process | 0.00533 | 0.084 |
| GO:0045087 | innate immune response | biological_process | 0.00553 | 0.0843 |
| GO:0016064 | immunoglobulin mediated immune response | biological_process | 0.0058 | 0.0844 |
| GO:0032945 | negative regulation of mononuclear cell proliferation | biological_process | 0.0058 | 0.0844 |
| GO:0050672 | negative regulation of lymphocyte proliferation | biological_process | 0.0058 | 0.0844 |
| GO:0002694 | regulation of leukocyte activation | biological_process | 0.00629 | 0.0875 |
| GO:0019724 | B cell mediated immunity | biological_process | 0.00629 | 0.0875 |
| GO:0070664 | negative regulation of leukocyte proliferation | biological_process | 0.00654 | 0.0898 |
| GO:0030098 | lymphocyte differentiation | biological_process | 0.0067 | 0.0907 |
| GO:0002339 | B cell selection | biological_process | 0.00727 | 0.0943 |
| GO:0042613 | MHC class II protein complex | cellular_component | 1.03E-09 | 9.91E-07 |
| GO:0042611 | MHC protein complex | cellular_component | 8.85E-08 | 1.42E-05 |
| GO:0098797 | plasma membrane protein complex | cellular_component | 0.000762 | 0.0349 |
| GO:0005771 | multivesicular body | cellular_component | 0.00167 | 0.0535 |
| GO:0035693 | NOS2-CD74 complex | cellular_component | 0.00243 | 0.0535 |
| GO:0000323 | lytic vacuole | cellular_component | 0.00282 | 0.0557 |
| GO:0005764 | lysosome | cellular_component | 0.00282 | 0.0557 |
| GO:0035692 | macrophage migration inhibitory factor receptor complex | cellular_component | 0.00485 | 0.0777 |
| GO:0005773 | vacuole | cellular_component | 0.00546 | 0.0843 |
| GO:0005770 | late endosome | cellular_component | 0.00592 | 0.0848 |
| GO:0002142 | stereocilia ankle link complex | cellular_component | 0.00727 | 0.0943 |
| GO:1990696 | USH2 complex | cellular_component | 0.00727 | 0.0943 |
| GO:0042605 | peptide antigen binding | molecular_function | 0.000958 | 0.0418 |
| GO:0001968 | fibronectin binding | molecular_function | 0.00153 | 0.0535 |
| GO:0003953 | NAD+ nucleosidase activity | molecular_function | 0.00243 | 0.0535 |
| GO:0042658 | MHC class II protein binding, via antigen binding groove | molecular_function | 0.00243 | 0.0535 |
| GO:0050135 | NAD(P)+ nucleosidase activity | molecular_function | 0.00243 | 0.0535 |
| GO:0061809 | NAD+ nucleotidase, cyclic ADP-ribose generating | molecular_function | 0.00243 | 0.0535 |
| GO:0003823 | antigen binding | molecular_function | 0.00272 | 0.0557 |
| GO:0046982 | protein heterodimerization activity | molecular_function | 0.00422 | 0.0744 |
| GO:0035718 | macrophage migration inhibitory factor binding | molecular_function | 0.00485 | 0.0777 |
